# Transcriptome-based Phylogeny of the Semi-aquatic Bugs (Hemiptera: Heteroptera: Gerromorpha) Reveals Patterns of Lineage Expansion in a Series of New Adaptive Zones

**DOI:** 10.1093/molbev/msac229

**Published:** 2022-10-21

**Authors:** David Armisén, Séverine Viala, Isabelle da Rocha Silva Cordeiro, Antonin Jean Johan Crumière, Elisa Hendaoui, Augustin Le Bouquin, Wandrille Duchemin, Emilia Santos, William Toubiana, Aidamalia Vargas-Lowman, Carla Fernanda Burguez Floriano, Dan A Polhemus, Yan-hui Wang, Locke Rowe, Felipe Ferraz Figueiredo Moreira, Abderrahman Khila

**Affiliations:** Institut de Génomique Fonctionnelle de Lyon, Université de Lyon, Université Claude Bernard Lyon1, Centre National de la Recherche Scientifique Unité Mixte de Recherche 5242, Ecole Normale Supérieure de Lyon, 69007 Lyon, France; Evolutionary Biology, Department of Ecology and Genetics, Uppsala University, SE-752 36 Uppsala, Sweden; Institut de Génomique Fonctionnelle de Lyon, Université de Lyon, Université Claude Bernard Lyon1, Centre National de la Recherche Scientifique Unité Mixte de Recherche 5242, Ecole Normale Supérieure de Lyon, 69007 Lyon, France; Laboratório de Biodiversidade Entomológica, Instituto Oswaldo Cruz, Fundação Oswaldo Cruz, RJ, 21040-900 Rio de Janeiro, Brazil; Institut de Génomique Fonctionnelle de Lyon, Université de Lyon, Université Claude Bernard Lyon1, Centre National de la Recherche Scientifique Unité Mixte de Recherche 5242, Ecole Normale Supérieure de Lyon, 69007 Lyon, France; Institut de Génomique Fonctionnelle de Lyon, Université de Lyon, Université Claude Bernard Lyon1, Centre National de la Recherche Scientifique Unité Mixte de Recherche 5242, Ecole Normale Supérieure de Lyon, 69007 Lyon, France; Institut de Génomique Fonctionnelle de Lyon, Université de Lyon, Université Claude Bernard Lyon1, Centre National de la Recherche Scientifique Unité Mixte de Recherche 5242, Ecole Normale Supérieure de Lyon, 69007 Lyon, France; Department of Ecology and Evolutionary Biology, University of Toronto, M5S 3B2 Toronto, Ontario, Canada; Center for Scientific Computing (sciCORE), University of Basel, 4056 Basel, Switzerland; Institut de Génomique Fonctionnelle de Lyon, Université de Lyon, Université Claude Bernard Lyon1, Centre National de la Recherche Scientifique Unité Mixte de Recherche 5242, Ecole Normale Supérieure de Lyon, 69007 Lyon, France; Institut de Génomique Fonctionnelle de Lyon, Université de Lyon, Université Claude Bernard Lyon1, Centre National de la Recherche Scientifique Unité Mixte de Recherche 5242, Ecole Normale Supérieure de Lyon, 69007 Lyon, France; Institut de Génomique Fonctionnelle de Lyon, Université de Lyon, Université Claude Bernard Lyon1, Centre National de la Recherche Scientifique Unité Mixte de Recherche 5242, Ecole Normale Supérieure de Lyon, 69007 Lyon, France; Laboratório de Biodiversidade Entomológica, Instituto Oswaldo Cruz, Fundação Oswaldo Cruz, RJ, 21040-900 Rio de Janeiro, Brazil; Department of Natural Sciences, Bishop Museum, 1525 Bernice St., Honolulu 96817, HI, USA; School of Life Sciences, Sun Yat-sen University, 135 Xingangxi Road, Guangzhou 510275, Guangdong, People’s Republic ofChina; Department of Ecology and Evolutionary Biology, University of Toronto, M5S 3B2 Toronto, Ontario, Canada; Laboratório de Biodiversidade Entomológica, Instituto Oswaldo Cruz, Fundação Oswaldo Cruz, RJ, 21040-900 Rio de Janeiro, Brazil; Institut de Génomique Fonctionnelle de Lyon, Université de Lyon, Université Claude Bernard Lyon1, Centre National de la Recherche Scientifique Unité Mixte de Recherche 5242, Ecole Normale Supérieure de Lyon, 69007 Lyon, France; Evolutionary Biology, Department of Ecology and Genetics, Uppsala University, SE-752 36 Uppsala, Sweden

**Keywords:** adaptation, adaptive zone, Gerromorpha, key innovation, phylogeny, semi-aquatic bugs

## Abstract

Key innovations enable access to new adaptive zones and are often linked to increased species diversification. As such, innovations have attracted much attention, yet their concrete consequences on the subsequent evolutionary trajectory and diversification of the bearing lineages remain unclear. Water striders and relatives (Hemiptera: Heteroptera: Gerromorpha) represent a monophyletic lineage of insects that transitioned to live on the water–air interface and that diversified to occupy ponds, puddles, streams, mangroves and even oceans. This lineage offers an excellent model to study the patterns and processes underlying species diversification following the conquest of new adaptive zones. However, such studies require a reliable and comprehensive phylogeny of the infraorder. Based on whole transcriptomic datasets of 97 species and fossil records, we reconstructed a new phylogeny of the Gerromorpha that resolved inconsistencies and uncovered strong support for previously unknown relationships between some important taxa. We then used this phylogeny to reconstruct the ancestral state of a set of adaptations associated with water surface invasion (fluid locomotion, dispersal and transition to saline waters) and sexual dimorphism. Our results uncovered important patterns and dynamics of phenotypic evolution, revealing how the initial event of water surface invasion enabled multiple subsequent transitions to new adaptive zones on the water surfaces. This phylogeny and the associated transcriptomic datasets constitute highly valuable resources, making Gerromorpha an attractive model lineage to study phenotypic evolution.

## Introduction

Key innovations enable access to new adaptive zones (see definition in (Volvenko 2022)) where lineages can diversify and occupy new niches (Simpson 1953; Mayr 1963; Schluter 2000; Rabosky 2017). These innovations have attracted much attention, because they are often linked to increased diversification (Liem 1973; Schluter 2000; Wagner and Lynch 2010; Wagner 2017; Ronco, et al. 2021). Spectacular examples of this process include the evolution of flight in bats and herbivory in insects. Understanding the processes underlying transitions to new adaptive zones and the consequences of these transitions on the evolutionary trajectory of lineages has greatly enriched our understanding of species diversification. These studies typically focus on the evolution and ecological function of traits or sets of traits that constitute the innovation and its consequences for diversity in the lineage. More recently, studies have begun to focus on the developmental genetics underlying these traits (Abzhanov, et al. 2004; Santos, et al. 2017).

Arthropods inhabit a great variety of habitats, from marine to freshwater, and from land to air. However, very few lineages permanently inhabit the air–water interface (e.g., whirligig beetles, fishing spiders, and water striders). This lifestyle may be limited by the constraints of fluid dynamics on remaining on the water surface and generating efficient thrust for movement on the fluid substrate (Andersen 1982; Hu and Bush 2010). Here we focus on water striders and relatives, also known as semi-aquatic bugs (Hemiptera: Heteroptera: Gerromorpha), which represent a group of insects characterized by their ability to live on the air–water interface (Andersen 1982). This group is a prominent example of a lineage that transitioned to a new adaptive zone, the water surface, and offers a unique opportunity to study the causes and consequences of this transition on species diversification. Since the initial transition to water surface habitats, the semi-aquatic bugs radiated into over 2,000 extant species occupying a vast array of niches ranging from rain puddles to ponds, streams, lakes, mangroves, and even the open oceans (Andersen 1982; Polhemus and Polhemus 2008).

The Gerromorpha are characterized by a large array of adaptive, and often conspicuous, traits associated with water surface locomotion. Layers of hydrophobic setae covering their legs sequester air, forming a cushion between the legs and the water, thus allowing the animals to avoid breaking surface tension (Andersen 1982; Gao and Jiang 2004; Hu and Bush 2010). Other traits include the elongation of the legs relative to terrestrial species that increases speed on the water (Crumière, et al. 2016). This change is characteristic of lineages that occupy marginal aquatic zones and that can walk both on water and on land. Other lineages have specialized in open water zones and propel their body by means of surface rowing (Andersen 1982; Crumière, et al. 2016). This derived mode of locomotion is tightly associated with a change in the body plan to a derived state where midlegs are longer and are primarily responsible for propulsion through simultaneous sculling motion, also known as the rowing gait (Andersen 1976). While the contribution of the set of adaptive traits to the initial transition to the water surface has been the subject of many studies (Andersen 1982; Gao and Jiang 2004; Hu and Bush 2010; Wang, et al. 2015), the consequences of this transition on the subsequent evolutionary trajectories and diversification of the lineage remain unclear.

The Gerromorpha also represents a prominent model for the study of sexual selection (e.g. Rowe, et al. 1994; Fairbairn and Preziosi 1996; Arnqvist 1997; Preziosi and Fairbairn 2000; Arnqvist and Rowe 2002a, b; Ronkainen, et al. 2010; Crumière and Khila 2019; Toubiana and Khila 2019; Toubiana, Armisén, Dechaud, et al. 2021). In many species, the mating system is characterized by sexual conflict, which is often manifested by rigorous pre-mating struggles where females resist costly mating attempts and males persist (Arnqvist 1989; Rowe 1994; Ronkainen, et al. 2010). These premating struggles have favored the repeated evolution of remarkable grasping structures in males and anti-grasping structures in females (Rowe, et al. 2006; Crumière, et al. 2019), which appear to coevolve antagonistically (Arnqvist and Rowe 2002a; Perry, et al. 2017; Crumière and Khila 2019). It is interesting that the characteristic sexual conflict in the mating system of many members of this group may itself have resulted from the transition to the two-dimensional water surface habitat, where females are relatively easy to detect, locate, and approach (Arnqvist 1997).

Previous works documented the developmental genetic mechanisms underlying appendage diversification in a selection of gerromorphans. The Hox gene *Ultrabithorax* has been key to increasing leg length and in changes in body plan associated with water surface locomotion and predation escape (Khila, et al. 2009, 2014; Refki, et al. 2014; Armisén, et al. 2015; Refki and Khila 2015). Interestingly, the same gene is involved in modifying male third legs into claspers under sexually antagonistic selection in the water strider *Rhagovelia antilleana* (Crumière and Khila 2019). In addition, we recently began to elucidate the developmental genetics of other sexually antagonistic traits consisting of modified antennae (Khila, et al. 2012) or other independent cases of male third leg modifications (Toubiana, Armisén, Dechaud, et al. 2021; Toubiana, Armisén, Viala, et al. 2021). However, the evolutionary history and the combined impact of water surface locomotion and sexual conflict on the diversification of the legs in water striders remain to be tested.

This rich natural history, phenotypic diversity, and the development of genetic tools, together with the invasion of the water surface habitat, provide an invaluable opportunity to study the patterns and processes underlying phenotypic evolution of lineages following the transition to new adaptive zones. A major constraint on this opportunity is the lack of a well-supported phylogeny of the whole infraorder, since those currently available have been recovered using a limited number of morphological characters and/or molecular markers (e.g., Andersen 1982; Andersen and Weir 2004; Damgaard 2008a). While the current phylogenies generally agree on the position of certain nodes at higher taxonomic levels, some lineages remain problematic or unresolved (Damgaard 2008a). In particular, the currently accepted higher classification of the group has inconsistencies at the levels of family and subfamily, and key taxa remain unassessed (Damgaard 2008a, 2012; Damgaard, et al. 2014; Román-Palacios, et al. 2020). These inconsistencies hinder reliable reconstructions of ancestral character states, thus limiting our understanding of the evolutionary history of this group.

In this work, we reconstructed the phylogeny of the infraorder using transcriptome-based genome-wide markers. This effort resolved inconsistencies and uncovered strong support for previously unknown relationships between some important taxa. We dated the phylogeny based on the available fossil record and reconstructed the evolutionary history of a set of adaptive characters associated with water surface locomotion, dispersal, transition from fresh to saline waters and sexual dimorphism. Our results reveal how the initial event of water surface invasion enabled multiple subsequent transitions to new adaptive zones and further diversification within the lineage.

## Results

### Transcriptome Assemblies and Gene Clustering

**Fig. 1.**
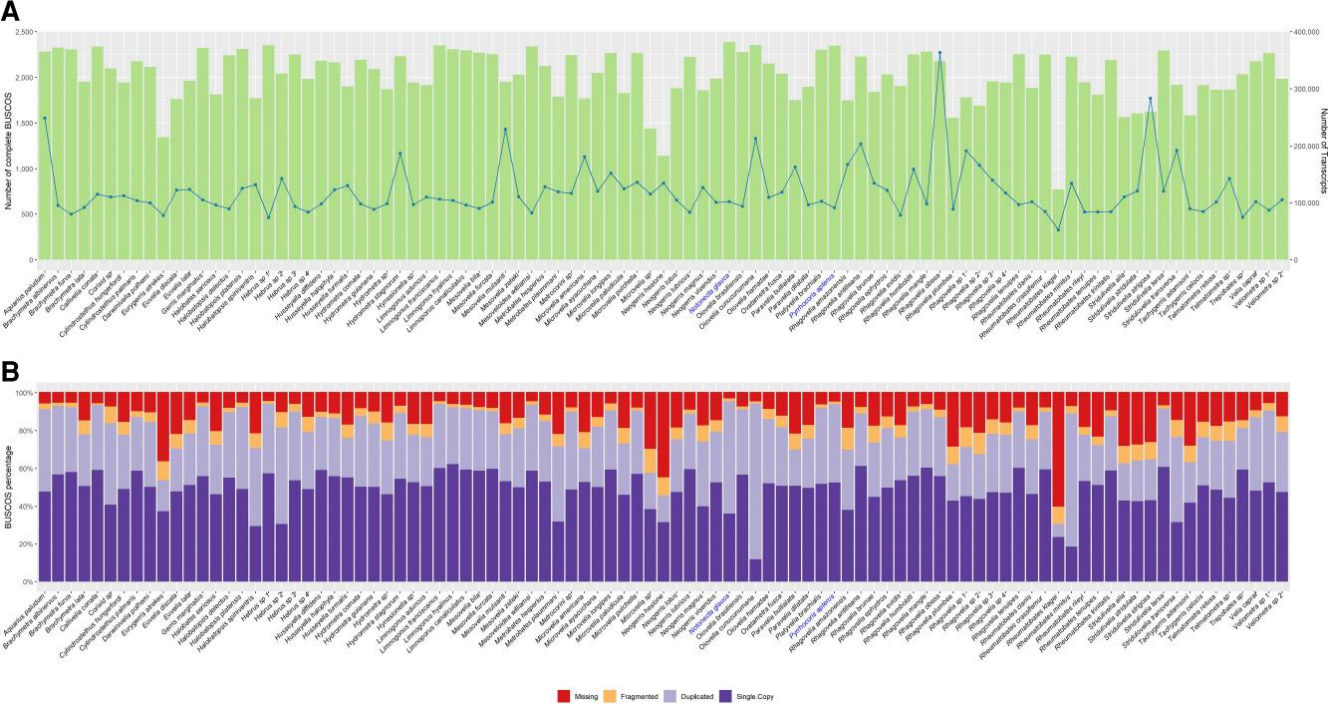
Abundance and completeness of transcriptomics datasets. (*A*) Green histogram: Number of complete BUSCOs (db10) found in the assembled transcriptome of each newly sequenced species (92 Gerromorpha and 3 outgroup species); Blue line: Number of de novo assembled transcripts by Trinity. (*B*) Breakdown of BUSCOs results percentages including complete (single-copy in dark purple and duplicated in light purple), fragmented in orange and missing in red. In both cases names of outgroup species (i.e., not Gerromorpha) were colored in blue.

We sequenced the transcriptomes of 95 species, of which 92 cover five out of the eight known families of Gerromorpha and 3 are outgroup species (supplementary table S1, Supplementary Material online). The number of Illumina reads obtained ranged between 43 million and 275 million (supplementary table S2, Supplementary Material online). Transcriptome assemblies using Trinity yielded between 52,462 and 363,535 transcripts with BUSCOs 5.2.2 completeness ranging from 30.7% to 95.1% (fig. 1 and supplementary table S2, Supplementary Material online). Nine additional transcriptomes from other Gerromorpha or closely related species were included from other sources (see Methods). We used three different methods to define clusters of orthologous genes (OrthoFinder (Emms and Kelly 2019), SiLiX (Miele, et al. 2011), and BUSCOs (Simao, et al. 2015; Waterhouse, et al. 2018)) and selected those with at least 80% of the species (4,181, 3,174 and 1,869, respectively). After testing for stationary, reversible, and homogeneous (SRH) consistency (Naser-Khdour, et al. 2019), a final list of 686, 851, and 528 gene clusters, respectively, was retained for phylogeny reconstruction using IQ-TREE and TNT (see Methods for more details).

### Recovering Gerromorpha Phylogeny Using Transcriptomic Markers

**Fig. 2.**
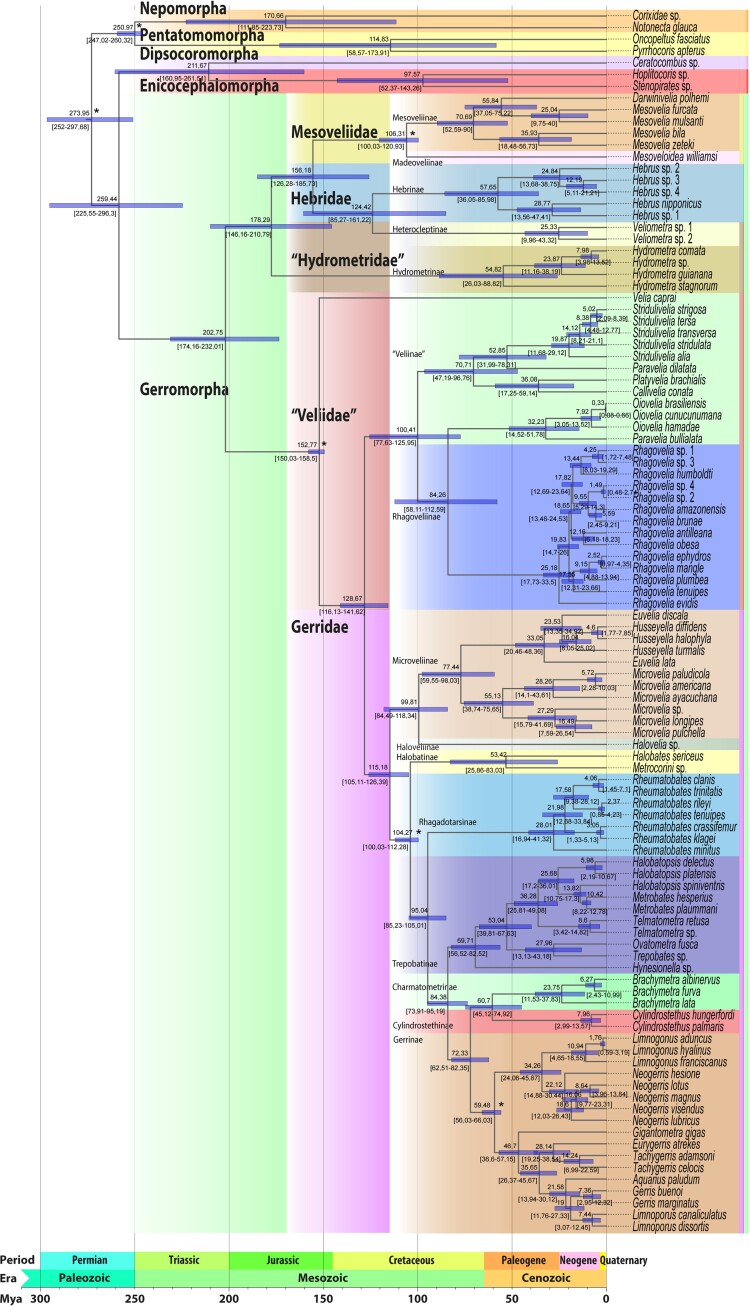
Dated phylogeny of Gerromorpha. Phylogenetic tree was reconstructed with IQ-TREE (Nguyen, et al. 2015) using Maximum-Likelihood (ML) on nucleotide sequence (position 1 + 2) from OrthoFinder clusters (Emms and Kelly 2019). Best partitioning scheme and substitution model for each partition were identified using ModelFinder (Kalyaanamoorthy, et al. 2017). Robustness was estimated from 1,000 bootstrap replicates using ultrafast bootstrap (UFBoot) approximation (Minh, et al. 2013) (more details in Methods section). Timescale was estimated from six calibration points (marked with an asterisk [*]). Estimated ages are indicated on each node and the 95% confidence interval is indicated with a blue box while confidence interval ages are presented between square brackets. Families and subfamilies are highlighted in various colors with names indicated. Paraphyletic taxa are indicated in quotation marks.

The infraorder Gerromorpha, as well as the families Mesoveliidae, Hebridae, and Gerridae, were all recovered as monophyletic (fig. 2). Hydrometridae (hereafter “Hydrometridae”) is however polyphyletic, because *Veliometra* (“Hydrometridae”: Heterocleptinae) is closer to *Hebrus* (Hebridae: Hebrinae) than to *Hydrometra* (“Hydrometridae”: Hydrometrinae). Because this is the first time that this relationship is recovered and we lack samples of other “Hydrometridae” genera except *Hydrometra*, we refrain from proposing any classification changes at this time. Mesoveliidae is sister to (Hebridae + *Veliometra*), the three are sister to *Hydrometra*, and the clade as a whole is sister to (Veliidae + Gerridae). Veliidae (from now on “Veliidae”) is also not monophyletic (paraphyletic), consisting of a succession of clades sister to Gerridae. The same is also true for the subfamily Veliinae (“Veliidae”), in which *Velia caprai* was recovered as sister to all other clades of (“Veliidae” + Gerridae), while a clade containing species of *Oiovelia* and *Paravelia* is sister to *Rhagovelia* (“Veliidae”: Rhagoveliinae), not to the rest of the subfamily. Furthermore, the clade (Microveliinae + Haloveliinae) is more closely related to Gerridae than to the other subfamilies of “Veliidae”. Within Gerridae in the current sense (e.g., Damgaard 2008b), Halobatinae was recovered as sister to all other subfamilies, and Rhagadotarsinae as sister to (Trepobatinae + [Gerrinae + {Charmatometrinae + Cylindrostethinae}]).

These observations indicate that Microveliinae and Haloveliinae are not veliids and were consistently recovered regardless of dataset and methods we used (supplementary fig. S1 and S2, Supplementary Material online), which leads us to propose transferring them to the family Gerridae. Even with the removal of these two subfamilies, the current classification of “Veliidae” remains highly artificial and in need of a thorough revision.

### Time of Divergence of the Main Clades of Gerromorpha

The split of Gerromorpha dated using six fossil calibration points obtained from 11 fossil records (supplementary table S3, Supplementary Material online) was estimated to have occurred during the Upper Permian about 259 million year ago (Mya) (95% CI 225.55–296.3 Mya) (fig. 2), consistent with other phylogenetic analyses (Wang, et al. 2016, 2021; Johnson, et al. 2018). The divergence of the major clades of Gerromorpha is estimated to have started during the mid-Triassic about 202 Mya (95% CI 174.16–232.01 Mya) (fig. 2) when the clade (Gerridae + “Veliidae”) split from (“Hydrometridae” + [Mesoveliidae + Hebridae + Heterocleptinae]) early during the evolution of Gerromorpha. Again, those relationships were found regardless of dataset and methods we used (supplementary figs. S3 and S4, Supplementary Material online).

This result is inconsistent with classic phylogenies using either only morphological characters or combined with few molecular markers that recovered (Gerridae + Veliidae) as a derived and later branching clade (Andersen and Weir 2004; Damgaard 2008a), but consistent with most recent phylogenetic analyses using larger datasets (Wang, et al. 2016, 2021; Johnson, et al. 2018). In this scenario, “Hydrometridae” split from ((Hebridae + Heterocleptinae) + Mesoveliidae) 178.29 Mya (95% CI 146.16–210.79 Mya), whereas (Hebridae + Heterocleptinae) split from Mesoveliidae about 156 Mya (95% CI 126.28–185.73 Mya). In turn, Heterocleptinae split from Hebrinae about 124.42 MYA (95% CI 85.27–161.22 Mya).

On the other side of the tree, the split between “Veliidae” (already excluding Microveliinae and Haloveliinae) and Gerridae took place during the Mesozoic (Lower Cretaceous), about 128 Mya (95% CI 116.13–141.32 Mya) (fig. 2). Finally, Microveliinae + Haloveliinae diverged from the other Gerridae subfamilies about 115 Mya (95% CI 105.11–126.39 Mya).

### Single Origin of Rowing as a Derived Mode of Locomotion in “Veliidae” and Gerridae

Ancestral character state reconstruction shows a significant increase in the ratio of midleg to body length in both males and females (supplementary figs. S6 and S9, Supplementary Material online) at the base of Gerromorpha coinciding with the transition to water surface habitats (Crumière, et al. 2016). The increase in length is more modest for the forelegs and hindlegs (supplementary figs. S5 –S8 and S10, Supplementary Material online), consistent with the primary role of the midlegs in generating propulsion even when the animals retain the ancestral walking gait (Crumière, et al. 2016). These data suggest that increased leg length is among the phenotypic changes associated with the acquisition of marginal water areas as a new adaptive zone.

**Fig. 3.**
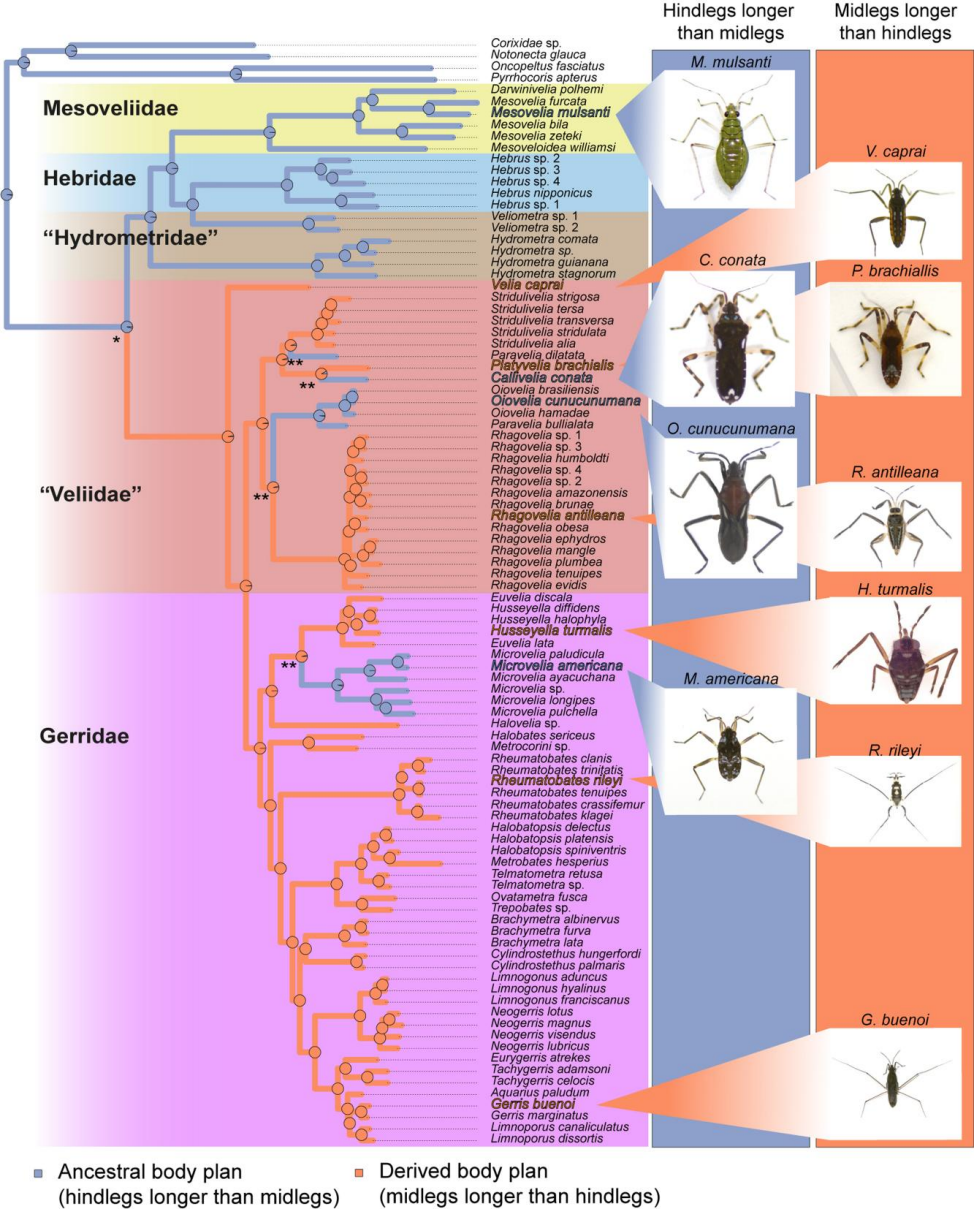
Ancestral character state reconstruction of relative leg length, with midlegs being the longest associated with rowing and hindlegs being the longest associated with walking (see Crumière, et al. (2016)). Reconstruction was performed with “phytools::make.simmap” R function using an equal-rates model (ER). Pies represent the probability of ancestral state. Images represent samples of the two relative leg length phenotypes and associated locomotion mode. Derived body plan (midlegs longer than hindlegs) event and transition to open waters is marked with an asterisk (*). Reversals to ancestral body plan (hindlegs longer than midlegs) are marked with a double asterisk (**).

Lineages of Gerromorpha that transitioned to open waters use the rowing gait, which is tightly associated with a novel body plan where midlegs are the longest (Andersen 1976; Hu, et al. 2003; Crumière, et al. 2016). We scored the state of body plan across our sample to reconstruct the evolutionary history of locomotion modes in Gerromorpha (fig. 3). We found that leg morphology associated with the walking gait is ancestral and that the switch to midlegs being longer evolved only once some 153 Mya in the common ancestor of (“Veliidae” + Gerridae) (fig. 3). Within this clade, we detected four independent reversals from the rowing morphology back to the walking morphology, three times in the “Veliidae” and once in the Microveliinae (fig. 3).

### Repeated Co-option of Male Hindlegs into Grasping Structures Through a Common Genetic Pathway

**Fig. 4.**
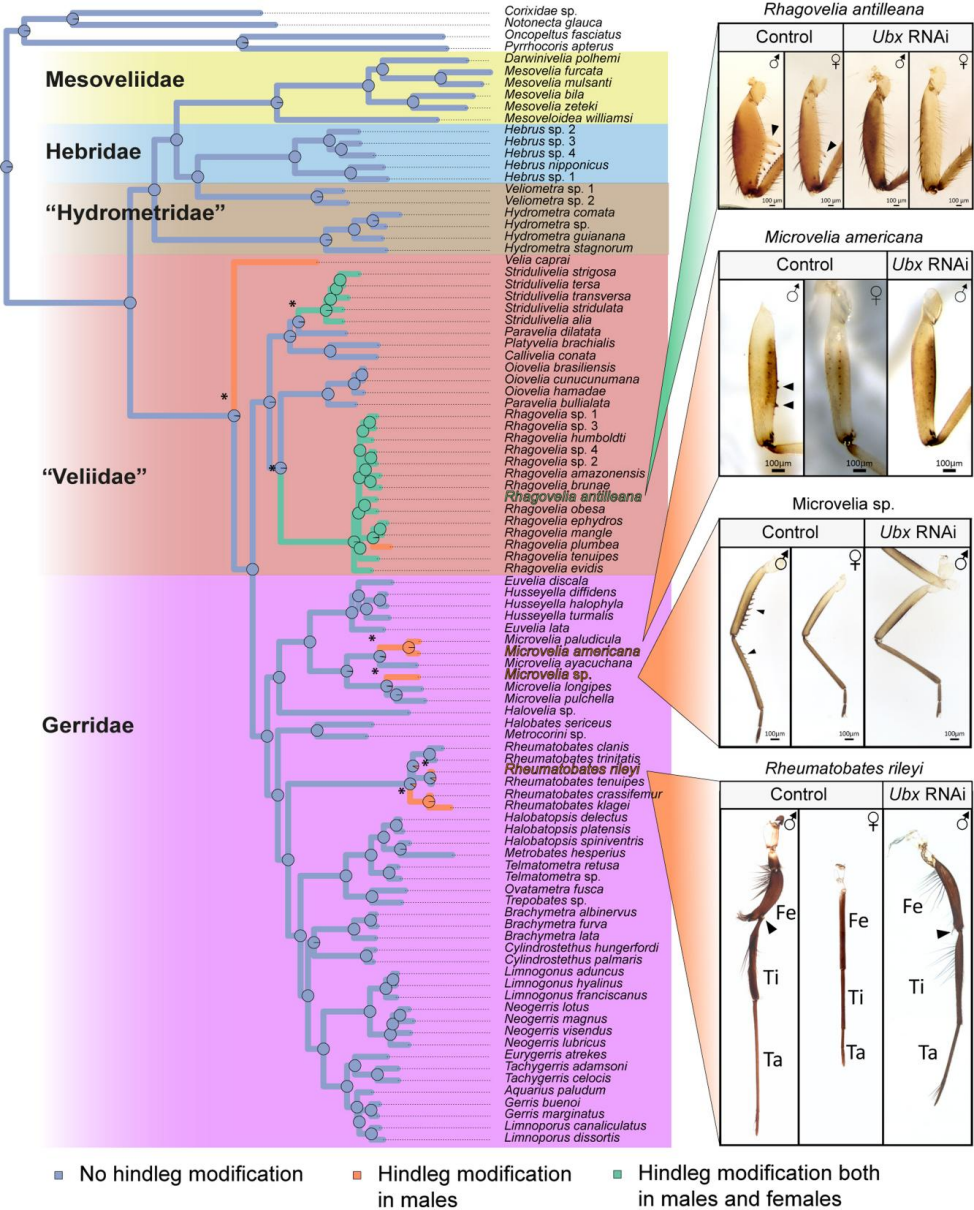
Ancestral character state reconstruction of male hindleg modifications into grasping traits driven by sexual conflict. Reconstruction was performed with “phytools::make.simmap” R function using a symmetrical model (SYM). Pies represent the probability of ancestral state. Images of four lineages that evolved male hindleg modifications, along with female hindleg comparisons, are represented. *Ubx* RNAi removes male modification in all lineages regardless of morphology or phylogenetic position. Independent leg modification events are marked with an asterisk (*).

The biomechanical requirements of movement on the water surface may have driven the evolution of relative leg lengths in the Gerromorpha (Andersen 1982; Khila, et al. 2014; Crumière, et al. 2016). Sexually antagonistic coevolution has also resulted in elaboration and dimorphism of these appendages, sometimes profound (Rowe, et al. 2006; Crumière, et al. 2019). Some of the most common grasping structures in males are the modifications of the appendages into clamps that increase male ability to overcome female resistance to mating. Modification of the mid- and forelegs to increase their utility as clamps are common in the veliids and gerrids, yet modification of the midlegs into claspers, the primary appendage for water surface locomotion, is rare. Phylogenetic reconstruction revealed that the elaboration of male hindlegs into clamps evolved at least seven times independently in our sample (fig. 4). In some genera such as *Rheumatobates* or *Microvelia*, male modifications of the hindlegs evolved multiple times within the genus and are represented twice in our sampling (fig. 4). This is an under-estimation, as Rowe and colleagues have shown that male modified hindlegs evolved four times independently within the genus *Rheumatobates* (Rowe, et al. 2006). The modifications resulted in either divergent (*Rheumatobates*) or convergent (e.g. *Microvelia, Rhagovelia, Stridulivelia*) morphologies. Among the seven lineages that independently evolved modifications of male hindlegs, the females of five do not bear any modifications, suggesting frequent resolution of intra-locus sexual conflict (Stewart, et al. 2010). In the two genera *Stridulivelia* and *Rhagovelia* (except *R. plumbea* in our sample), females retain hindleg modifications albeit to a lesser degree than males, suggesting that genetic regulation underlying these modifications is common between the sexes and may constrain the evolution of sexual dimorphism in these two taxa (fig. 4). Interestingly, modified male hindlegs evolved only after the evolutionary elongation of the midlegs associated with rowing—a mode of locomotion that relies primarily on the midlegs (Andersen 1976; Hu et al. 2003; Crumière et al. 2016). These results suggest that the primary use in locomotion of the midlegs may constrain their elaboration, whereas hindlegs (and forelegs or even antennae) are free to evolve in response to sexual conflict.

The Hox gene *Ultrabithorax* (*Ubx*) is involved in the increase of the absolute length of both mid- and hindlegs in the walking species (Khila, et al. 2014; Refki, et al. 2014), in reversing the relative lengths of the mid- and hindlegs (and their segments) in the rowing species (Khila, et al. 2009, 2014; Refki, et al. 2014; Armisén, et al. 2015), and for the modifications of male hindlegs into clamps in the water strider *Rhagovelia antilleana* (Crumière and Khila 2019). Furthermore, *Ubx* depletion removes the modifications from female hindlegs, thus confirming that *R. antilleana* males and females share the genetic basis of hindleg modifications (Crumière and Khila 2019) (fig. 4). To test whether Ubx was involved in the other independent events of male hindleg modifications, we depleted *Ubx* transcripts using RNAi in three additional species, each representing an independent event of hindleg modifications in males (*Microvelia americana*, *Microvelia* sp. [new] and *Rheumatobates rileyi*). *Rheumatobates rileyi* males present hindleg modifications that are deeply divergent from *Rhagovelia antilleana* and *Microvelia* spp. (fig. 4). Strikingly, *Ubx* depletion across these three additional species resulted in the loss of the modifications of male hindlegs regardless of the morphology of the leg or the phylogenetic position of the bearing species (fig. 4). These findings support the repeated and independent use of *Ubx* during the evolution of male sexually antagonistic traits driven by sexual conflict throughout the evolutionary history of the Gerromorpha.

### Independent Transitions From Fresh to Saline Waters

**Fig. 5.**
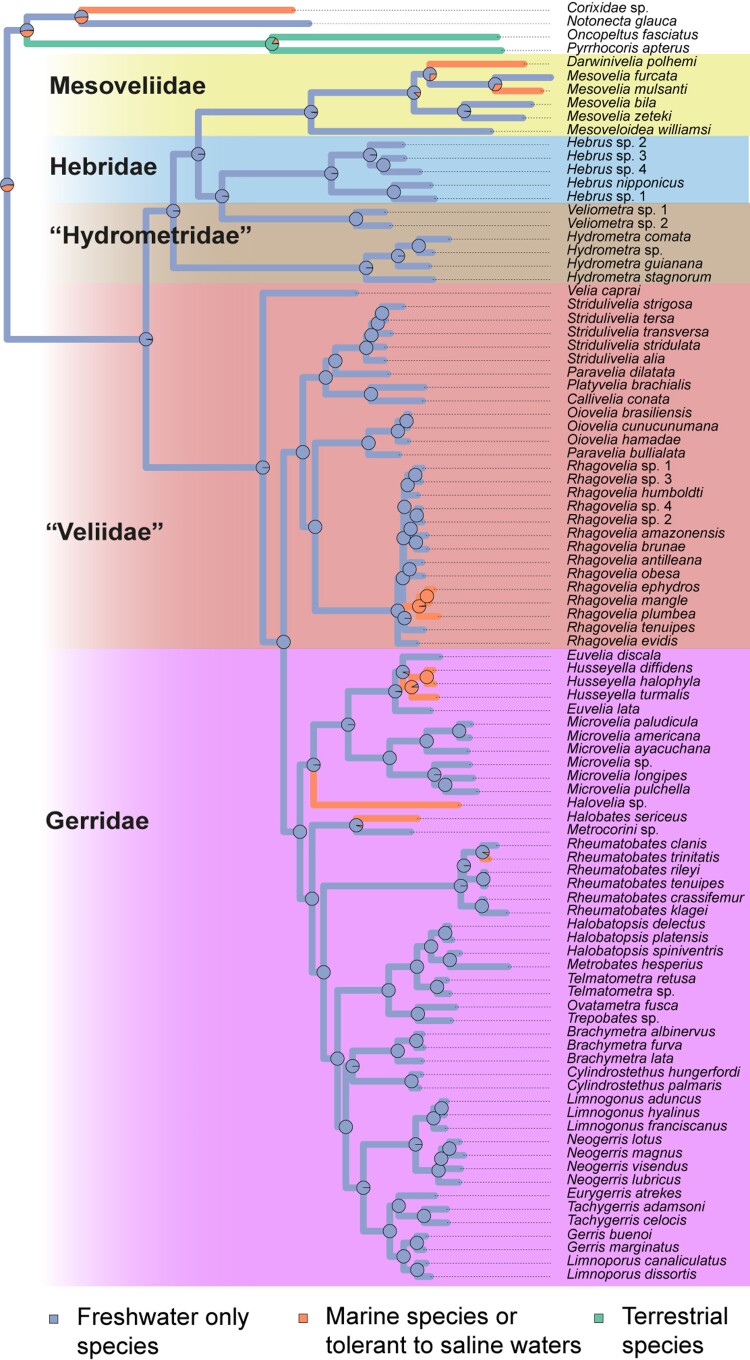
Ancestral character state reconstruction of habitat preference with regard to salinity. Reconstruction was performed with “phytools::make.simmap” R function using a symmetrical model (SYM). Pies represent the probability of ancestral state. Independent transitions marked with an asterisk (*).

Out of the million or so described species of insects, the Gerromorpha have been astonishingly successful in colonizing saline water habitats, including the open oceans (Andersen 1982, 1999; Cheng 1989; Ryland and Tyler 1989; Ikawa, et al. 2012). Various lineages occupy habitats with different degrees of salinity ranging from brackish to high salinity seawaters. The salty *versus* freshwater preference covers both micro- and macro-evolutionary scales (Andersen 1999). In some genera, both salty and freshwater populations of the same species can be found, but some lineages either at the family level or at the genus level are exclusively marine (Andersen 1999; Ikawa, et al. 2012). Our sampling included 10 lineages represented by 10 species out of 97, which were known to inhabit and were collected in marine or brackish environments (fig. 5). The ability to occupy salty water bodies evolved early in the Gerromorpha during the Cretaceous in the Mesoveliidae, “Veliidae”, and Gerridae (figs. 2 and 5). Our character state reconstruction revealed that, of these 10 lineages, seven have independently transitioned from fresh to saline environments (fig. 5). Some of these transitions occurred below the genus level, as is the case for *Rheumatobates, Rhagovelia,* or *Mesovelia* (fig. 5). Other events characterize entire lineages; such is the case for *Halobates* or *Halovelia*. Among these transitions, some lineages specialize in mangroves where water salinity fluctuates from almost fresh to entirely marine within the same day. These include *Rheumatobates trinitatis*, *Husseyella halophyla*, *Rhagovelia mangle,* and *Rhagovelia ephydros*. This indicates that these lineages are versatile and have evolved phenotypic plasticity that allows them to expand their habitat range to environments with short-term fluctuations in salinity. Other lineages, including *Halobates* or *Halovelia*, are exclusively marine, except for secondary reversions to the freshwater life (Román-Palacios, et al. 2020), indicating that they have become specialized on salt water (Cheng 1976; Sekimoto, et al. 2014).

### Evolution of Wing Polymorphism

**Fig. 6.**
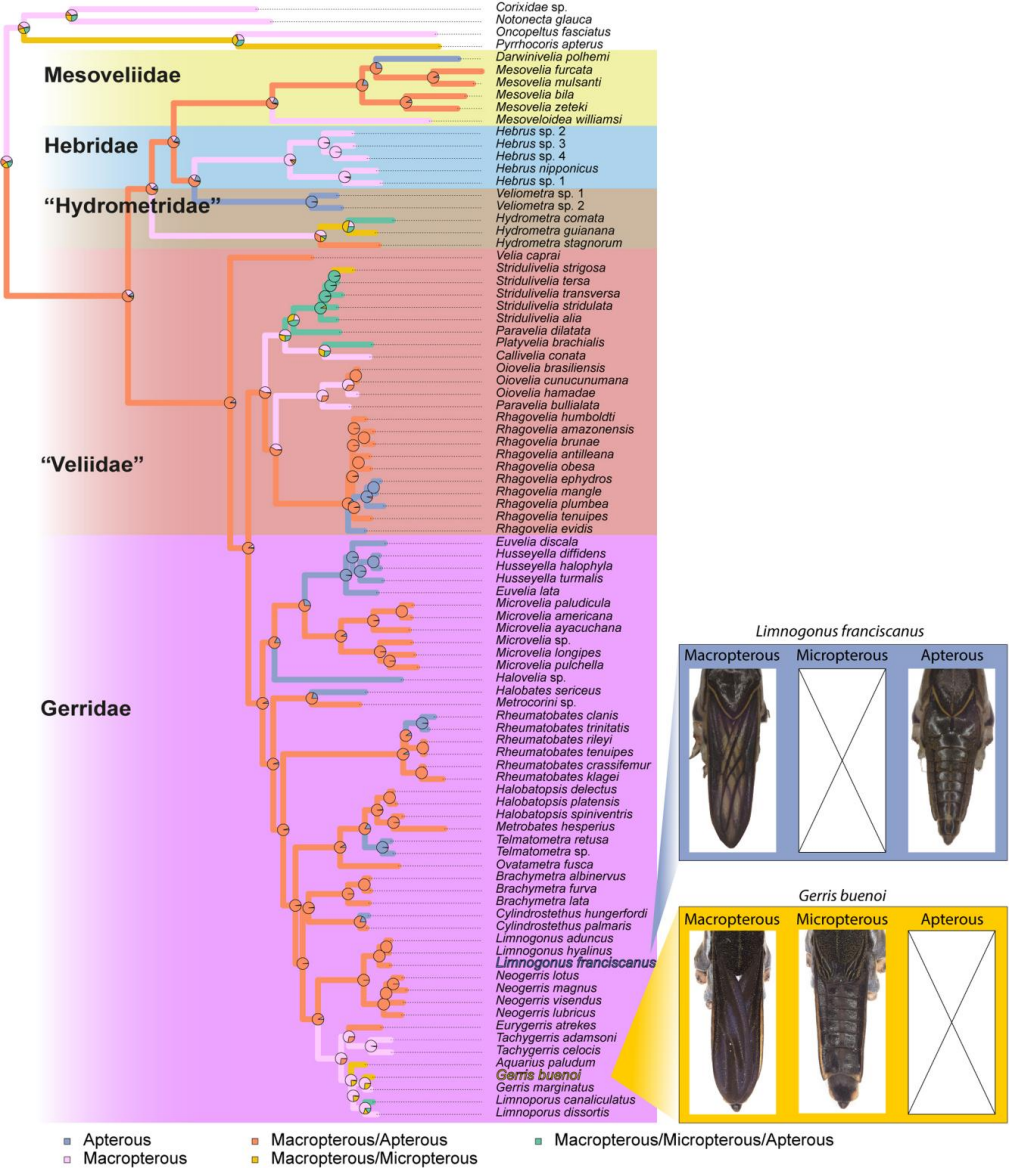
Ancestral character state reconstruction of wing polymorphism. Reconstruction was performed with “phytools::make.simmap” R function using a symmetrical model (SYM). Pies represent the probability of ancestral state. Images represent examples of wing polymorphism in two species: macropterous and apterous in *Limnogous franciscanus*, and macropterous and micropterous in *Gerris buenoi*. Apterous, No wing morph; Micropterous, Short wing morph; Macropterous, Long wing morph.

Wing polymorphism is widespread in the Gerromorpha and a variety of wing morphs, from complete absence (apterous) to long wing morphs (macropterous) have been described both within and across species (fig. 6). An intermediate morph (brachypterous) has also been described in some species, but for simplicity this morph was not included in the reconstruction as amongst our sample it only occurs in few species. Among the species we sampled, 16 have exclusively long wings, 19 exclusively no wings, and the remaining species are polymorphic. *Cylindrostethus hungerfordi* was herein considered as entirely apterous, because only one macropterous specimen was recorded in the literature (Nieser 1970), which pends verification. The complete loss of wings is characteristic to marine species, which are represented in our sampling by the genera *Darwinivelia*, *Halovelia,* and *Halobates* (Andersen 1982; Polhemus and Polhemus 2008). Among the 91 Gerromorpha species included in our phylogeny for which we had reliable wing morph data, we reconstructed seven independent events of wing loss, either at the genus level such as in *Veliometra*, *Darwinivelia*, *Halovelia*, *Halobates,* and *Euvelia/Husseyella*, or at the species level such as in *Rhagovelia*, *Rheumatobates,* and *Cylindrostethus*, where four, two and one species lost macropterous morphs, respectively (fig. 6). Wing polymorphism, i.e. the presence of more than one wing morph in a single species, evolved twice independently within Mesoveliidae and in the common ancestor of (“Veliidae” + Gerridae) (fig. 6). Within the latter clade, the complete loss of wings evolved seven times and regains of a unique macropterous wing morph at least five times (fig. 6). These data suggest that the evolution of wing polymorphism is a dynamic process potentially influenced by the large heterogeneity of habitats, in terms of stability and seasonality, and the wide geographical distribution that these insects have occupied (Spence 1989; Harada and Numata 1993; Spence and Anderson 1994; Harada and Spence 2000; Fairbairn and King 2009).

## Discussion

Using hundreds of molecular markers, we generated a new phylogeny of the Gerromorpha that resolved inconsistencies and revealed that the evolutionary history of the lineage is rich in terms of transitions and expansions into new adaptive zones, enabled by important phenotypic changes to their appendages in association with new modes of locomotion on water. The multiple subsequent transitions to new adaptive zones may have been contingent on the initial event of water surface invasion common to all Gerromorpha.

### Evolutionary History of Gerromorpha

The results from our divergence time analysis suggest that the origin of Gerromorpha dates between Upper Triassic and Lower Triassic as suggested by fossil data reinterpretation (Damgaard 2008a, b) and similar to other recent phylogenomic analysis (Johnson, et al. 2018; Wang, et al. 2021). Our phylogenomic approach using a large number of genetic markers, instead of classical ribosomal DNA phylogenies combined with morphological traits (Damgaard 2008a, b), provided three major outcomes. First, we could clearly define “Hydrometridae” as sister to (Mesoveliidae + Hebridae) split. This result contrasts with earlier analysis that placed Mesoveliidae as the earliest branching clade within Gerromorpha and sister to all other families of the infraorder (Damgaard 2008a, b), but is consistent with the latest reconstructions for true bugs and hemiptera (Johnson, et al. 2018; Wang, et al. 2021). Second, we found that (“Veliidae” + Gerridae) diverged early in the evolution of Gerromorpha and is sister to (“Hydrometridae” + [Mesoveliidae + {Hebridae + Heterocleptinae}]) rather than a later branching clade (Damgaard 2008a, b). These two results are inconsistent with the widely accepted hypothesis and our own previous results (Crumière, et al. 2016; Santos, et al. 2017; Vargas-Lowman, et al. 2019). However, they are not unexpected, because: 1) fossil specimens attributed to the families Hydrometidae and Veliidae first appeared at a similar age, while records for Hebridae are much more recent; 2) it is possible that our increased number of species in the families “Hydrometridae”, Mesoveliidae and Hebridae allowed us to avoid long branch attraction artifacts that might be present in our previous phylogenetic reconstructions when only one representative for each family was used; and 3) phylogenetic analyses focused on other heteropterans already suggested a similar evolutionary scenario for Gerromorpha (Johnson, et al. 2018; Wang, et al. 2021). Finally, our phylogenetic reconstruction supports the hypothesis that Microveliinae and Haloveliinae are subfamilies of Gerridae instead of “Veliidae”. The sister relationship between Gerridae and (Microveliinae + Haloveliinae) had already been detected by Damgaard (2008a), who did not propose any classification changes. The whole clade was recovered here with full support and has two strong morphological synapomorphies. The first is the fusion of tarsomeres I and II in all legs that results in the tarsal formula 2:2:2 in Haloveliinae and Gerridae, which is further reduced to 1:2:2 by the fusion of all foreleg tarsomeres in Microveliinae (Andersen 1982). The second is the presence of a fecundation pump on the female gynatrial complex, which is believed to assist in the ejection of sperm through the apical part of the fecundation canal and into the lumen of the common oviduct prior to the fertilization of the passing egg (Andersen 1982).

### Diversification of the Legs in the Gerromorpha

The unique event of transition to water surface locomotion was accompanied by multiple phenotypic changes associated with exploiting surface tension, and generating efficient thrust on water (Hu and Bush 2010), such as increased density of hydrophobic bristles (Andersen 1976). We previously hypothesized that locomotion on water favors increased speed, which requires changes in locomotion parameters such as increased leg length and stroke frequency (Crumière, et al. 2016). This change was brought about through a gain of a new expression domain of the gene *Ubx* in the midlegs, in addition to its ancestral expression in the hindlegs (Khila, et al. 2009; Refki, et al. 2014). This role of Ubx had been possible after the initial gain of expression in the midlegs at the base of Gerromorpha, which enabled the increase in midleg length. The current ancestral character state reconstruction shows that leg length increased significantly at the base of the phylogeny, coinciding with the transition to the water surface (supplementary figs. S5 to S10, Supplementary Material online). Ancestral leg length relationship with hindlegs longer than midlegs was retained in, or regained by, lineages occupying the ancestral adaptive zone at the margin of water bodies (fig. 3). The (“Veliidae”+Gerridae) split early during the diversification of the infraorder and the split coincided with the evolution of rowing, enabling access to yet another adaptive zone composed of open waters (fig. 3). It appears that this new zone had many open niches as these two families now account for about 80% of all Gerromorpha (Polhemus and Polhemus 2008). The early change to the rowing body plan (fig. 3) brought about deep changes in locomotion behavior (Andersen 1982; Crumière, et al. 2016). Rowing gerromorphans generate fast and efficient thrust with significantly low frequency of strokes compared to walking Gerromorpha (Crumière, et al. 2016). This change is thought to allow these animals to sustain fast and efficient movement and expand into the vast surfaces of open water zones ranging from ponds to oceans (Cheng 1976; Andersen 1999).

Subsequent to the acquisition of rowing, certain taxa further evolved evolutionary innovations that allowed the transition to numerous other new niches. The family “Veliidae” contains about 60 genera and some 900 species, and the genus *Rhagovelia* alone accounts for almost half of the species count of the entire family (over 400 species) (Andersen 1982; Polhemus and Polhemus 2008). *Rhagovelia* spp. use rowing and all members of the genus possess a plumy fan on each midleg that allows generating efficient locomotion on fast flowing streams (Andersen 1982). The plumy fans are exclusive to the genus *Rhagovelia* and their evolution is likely to have enhanced their ability to survive in these fast-flowing streams. Four lineages (*Oioivelia*, *Paravelia*, *Callivelia,* and *Microvelia*) made the move back from the rowing to the walking body plan, and also back to the margins of water bodies (fig. 3) (Andersen 1976; Crumière, et al. 2016). Interestingly, among these lineages, *Oiovelia* became specialized in foamy habitats that form on the margins of streams, typically trapped by fallen tree trunks or other obstacles (Rodrigues, et al. 2014). Therefore, phenotypic changes, and the associated genotypic changes, not only can allow lineages to acquire new adaptive zones, but access to these also opens the possibility for further transition in a succession of niches where each new transition is facilitated by the previous.

In our character state reconstruction, it is noticeable that the modifications of male hindlegs into grasping structures evolved multiple times independently but exclusively in the lineages that diverged after the acquisition of rowing as a mode of locomotion (fig. 4). It is possible that the evolution of rowing, which almost exclusively relies on the midlegs to generate movement (Andersen 1976; Hu, et al. 2003; Crumière, et al. 2016), may have reduced constraints on the shape of the hindlegs due to biomechanical and hydrodynamics requirements no longer imposed by the ancestral walking mode. This change of functional emphasis on the role of the hindlegs may have freed these structures to evolve under sexual selection pressures. Coincident with the move to this two-dimensional habitat are high intersexual interactions that may increase sexual conflict, which in turn favors the evolution of male grasping traits such as modified hindlegs (Rowe, et al. 1994; Arnqvist 1997).

A characteristic of all Gerromorpha is the expansion of the domain expression of the Hox gene *Ultrabithorax* to cover both the midlegs and the hindlegs (Khila, et al. 2014; Refki, et al. 2014). It is possible that this initial genetic change enabled the midlegs to function as oars in association with water surface locomotion. It is also possible that this change provided the developmental genetic context for further modification of the legs, particularly the hindlegs, under sexual conflict again in association with Ubx function. The finding in four tested lineages, out of seven, that Ubx is always required for male hindleg modification indicates that few developmental genetic paths may be available for sexual conflict to drive male modifications. However, while the modifications are highly similar in species that possess spines (*e.g. Rhagovelia* and *Microvelia*), they are divergent from others (*Rheumatobates* in particular) where the elaborations involve deeper changes in the shape of the segments and the presence of sets of large setae along the proximo-distal axis. This is an indication that the developmental genetic programs controlled by Ubx might diverge among these lineages, resulting in phenotypic differences of the modifications of male legs.

### Specialization in Saline Habitats and Dispersal Through Flight

In our reconstruction, the ancestral habitat of the Gerromorpha is freshwater, which is consistent with previous reconstructions (Cheng 1976; Andersen 1999). Within Gerromorpha, the transition to saline water evolved throughout the phylogeny in at least seven independent instances out of the ten we sampled (fig. 5). Lineages transiting from freshwater to salty habitats faced a significant environmental change that is expected to affect fluid balance and concentration of electrolytes, which in turn is expected to pose significant challenges to osmoregulation. Common garden experiments testing tolerance to change in salinity revealed that many marine and freshwater lineages die quickly, with spasms and other signs of disrupted osmoregulation, when exposed to fresh and salty waters, respectively (Kishi, et al. 2006). The lineages that have made this transition must have undergone, independently, a series of physiological changes to regulate body fluid homeostasis. The nature of osmotic adaptation to brackish and saline water and the genetic mechanisms underlying these adaptations remain unknown in Gerromorpha.

Wings in Gerromorpha are important for dispersal through flight. Dispersal however is heavily influenced by environmental factors. In species with multiple wing morphs, population composition will depend on a combination of both genetic factors and the environment (Järvinen and Vepsäläinen 1976; Roff 1986; Simpson, et al. 2011), such as population density (Harada, et al. 1997; Harada and Spence 2000), food scarcity (Harada and Nishimoto 2007), salinity (Kishi, et al. 2007), temperature (Harada, et al. 2011), dryness (Harada 1998), photoperiod (Harada and Numata 1993), and habitat state (Cunha, et al. 2020). Wing development is an energy consuming process that is known to tradeoff with fertility (Hyun and Han 2021) and the presence of two or more wing morphs is widespread in Gerromorpha. Apterous, micropterous, and brachypterous individuals can be considered as a single functional category with the inability to disperse by means of flight. We found that the presence of multiple wing morphs is ancestral in Gerromorpha, with highly dynamic patterns of gains and losses as the lineages diversified within the various aquatic habitats (fig. 6). Interestingly, the complete loss of wings, and therefore the ability to disperse, is tightly associated with the transition to salty water bodies, as there is an over-representation of wing loss in halophilic species (Cheng 1976; Andersen 1982). The loss of wings has been linked to the stability of marine water environments, which may have favored investment in reproduction rather than dispersal. Nevertheless, some freshwater lineages, such as *Veliometra* and *Euvelia*, are also exclusively wingless, raising the question as to what characteristics of their habitat or reproductive behavior may have driven wing loss.

## Conclusions

Water striders and relatives (Hemiptera: Heteroptera: Gerromorpha) provide a great opportunity to study the patterns and processes underlying species diversification following the conquest of new adaptive zones. The new phylogeny allowed us to illustrate how multiple subsequent transitions to new adaptive zones may have been contingent on the initial adaptation to water surface life that is shared by all Gerromorpha. This adaptation, which occurred 259 Mya, a unique event of transition to water surface locomotion was accompanied by multiple phenotypic changes associated with exploiting surface tension. This first event was followed about 202 Mya by a split whereby a set of Gerromorpha lineages evolved rowing as novel mode of locomotion and transitioned to live in open water zones. Our results show that the evolution of this new mode of locomotion, and its associated body plan, occurred earlier than previously thought, suggesting a rapid diversification of the Gerromorpha to occupy new habitats from marginal shore waters to open oceans. A particular genus, *Rhagovelia*, which accounts for almost half of the Veliidae, evolved plumy fans that enhance locomotion in fast-flowing streams. We mapped the divergence of *Rhagovelia* to about 84 Mya followed by an impressive radiation with over 400 extant *Rhagovelia* spp. that specialize in fast flowing streams. Other processes, including sexual selection and phenotypic plasticity, have contributed to the diversification of the Gerromorpha. Our phylogenetic reconstruction show male evolved sexually antagonistic traits in the form of modified hindlegs at least seven times independently in species where locomotion relies primarily on the midlegs. We propose that the change in locomotion mode freed the hindlegs from their primary locomotory role allowing their modification under sexual conflict. Moreover, our gene function analyses identified the gene *Ubx* as a primary developmental player involved in the evolution of male modified hindleg regardless of their morphology or the phylogenetic position of the lineage that carries them. Finally, further independent adaptations have been important in the diversification of the Gerromorpha including transitions from freshwater to saline habitats and multiple events of gains and losses of wing polymorphism.

From a methological point of view, next-generation sequencing technologies, by decreasing costs and increasing the amount of information generated have been key to our ability to reconstruct the phylogenetic history of previously understudied lineages. The use of transcriptome datasets is even more affordable and realistic as whole genome sequencing of a large number of species is still costly and requires accurate genome annotation. Drawbacks of phylotranscriptomics based on next-generation sequencing include difficulties to obtain complete gene sequences, limited data for weakly expressed or non-expressed genes and uncertainties in gene number mainly due to the presence of multiple isoforms. However, many of these potential problems can be mitigated with a proper design of sampling, good specimen preservation, enough sequencing depth, and third-generation sequencing technologies. Transcriptome-based phylogenomics have been already used with success in the study of Hemiptera (Johnson, et al. 2018), true water bugs (Wang, et al. 2021) and, as presented here, in the semi-aquatic bugs. This approach has been critical in accurately rebuilding the phylogeny and allowed a reliable mapping and reconstruction of the evolutionary history of traits associated with their diversification. The data presented here constitute a valuable resource to the community interested in the study of phenotypic evolution.

## Methods

### Sample Collection and Culture

Natural populations were collected mainly during fieldwork in French Guyana and Brazil (supplementary table S1, Supplementary Material online). Additional samples were obtained from short trips to Canada and USA or from personal communication. Field samples were classified and stored in individual tubes per species in RNA*later*™ Stabilization Solution (Thermo Fisher Scientific). When possible, lab populations were generated from this initial natural population. The bugs were isolated by species and maintained in a bug room at 25 °C and 50% humidity in water tanks and fed on crickets. RNA was extracted either from lab populations or from natural population samples if lab breeding was unsuccessful.

### Taxonomy

Specimen identification was performed based on morphology using (Polhemus 1997; Moreira, et al. 2018) and by comparison with original descriptions, available redescriptions, and type or reference material previously deposited in collections accessible to the authors.

### Transcriptome Sequencing and Assembly

Poly-A RNA was isolated from embryos, nymphs and/or adults of 92 Gerromorpha, two Notonectidae and one Pyrrhocoridae (supplementary table S1, Supplementary Material online). Total RNA was extracted using TRIzol (Invitrogen) according to manufacturer's protocol. A first batch of libraries were sequenced using Hiseq 2500 paired end 100 nucleotides while a second batch was sequenced using Hiseq X ten paired end 150 nucleotides (supplementary table S1, Supplementary Material online). Transcriptomic reads were assembled using Trinity 2.5.1 (Grabherr, et al. 2011) and long ORFs with minimum protein length of 100 aa were extracted using TransDecoder 3.0.1 (Hass and Papanicolau 2013). Transcriptome completeness was assessed using the 2,510 BUSCOs in OrthoDB v10 Hemiptera (fig. 1, supplementary table S2, Supplementary Material online). To reduce computational burden, we used CD-HIT (Li and Godzik 2006; Fu, et al. 2012) with a sequence identity threshold of 0.995 and a word size of 5 to reduce the number of redundant transcripts.

### Other Transcriptomes

In addition to our 95 newly sequenced species, we included in our analyses the transcriptomes of six additional Gerromorpha as well as three close species available. *Limnoporus dissortis* (Gerromorpha) transcriptome was sequenced using 454 technology (Armisén, et al. 2015). *Gerris buenoi* (Gerromorpha) transcriptome was obtained from its annotated gene set (Armisén, et al. 2018). The transcriptomes of *Gigantometra gigas* (Gerromorpha), *Hebrus nipponicus* (Gerromorpha), and *Stenopirates* sp. (Encicocephalomorpha) were sequenced independently using Illumina Hiseq 2000 paired end 90 nucleotides, while *Halovelia* sp. (Gerromorpha) was sequenced using Illumina Hiseq 2000 paired end 150 nucleotides (Wang, et al. 2016). Finally, *Oncopeltus fasciatus* (Pentatomomorpha) gene transcripts were obtained from official gene set v1.1 (Panfilio, et al. 2019). *Hoplitocoris* sp. (Enicocephalomorpha), and *Ceratocombus* sp. (Dipsocoromorpha) transcriptomes were downloaded from (Johnson, et al. 2018). As with newly sequences transcriptomes, we extracted long ORFs with TransDecoder 3.0.1 (Hass and Papanicolau 2013) and filtered with CD-HIT (Li and Godzik 2006; Fu, et al. 2012) to create a reduced dataset and limit the computational burden.

### Contaminant Removal

A list of 73,737,464 sequence identifiers (gi) for archaea, bacteria, other, unclassified, and viruses, were e-fetched from NCBI database to detect potential contaminants. A second list of potential contaminant sequences from *Gryllus* and *Acheta* (34,738) was created because manual checks of first gene clusters detected traces of their ribosomal RNA in some samples. The origin of this contamination is brown crickets used as a food source for all Gerromorpha species bred in the lab. We searched potential contaminant from each source independently using BLASTN (Altschul, et al. 1990, 1997) (option: -db nt -gilist contaminant.txt -dust yes -evalue 1e-5 -max_target_seqs = 1000). We then recovered the id of any hit with an identity >90 and coverage >25% (for archaea, bacteria, other unclassified and viruses) and a minimum hit identity of 90 for *Gryllus* and *Acheta* (no minimum coverage). A total of 45,453 transcripts amongst all the species were identified as potential contaminants in CD-HIT reduced dataset (40,964 for archaea, bacteria, other unclassified and viruses and 4,489 for *Gryllus* and *Acheta*) and removed from further analysis.

### Orthologous Gene Clusters

We generated three different datasets of orthologous genes using OrthoFinder (Emms and Kelly 2019), SiLiX (Miele, et al. 2011), and BUSCOs (Simao, et al. 2015; Waterhouse, et al. 2018).

OrthoFinder: All transcriptomic sequences were used as input (option -f). Hierarchical Ortho Groups (HOGs) were recovered and those not containing at least 80% of the 104 species filtered out. The 4,181 remaining clusters (out of 181,933) were used as input clusters in the next steps.

SiLiX: Transcripts from coding proteins were aligned all-against-all using BLASTP (Altschul, et al. 1990; 1997) masking low complexity regions and threshold of 1e-5. Output results were then clustered for homologous sequences using the software package SiLiX 1.2.11 which implements an ultra-efficient algorithm based on single transitive links with alignment coverage constraints (Miele, et al. 2011). We ran SiLiX using stringent values of minimum 70% of identity and 80% overlap to define the clusters (options: -i 0.7 -r 0.8 -l 100 -m 0.5). The 3,258,665 protein-coding transcripts from all species yielded a total of 1,702,195 clusters, including singletons. Clusters where then filtered to remove those that fail to contain at least 80% of the species. A total of 3,174 clusters were retained. Nevertheless, while OrthoFinder applies a phylogenetic reconstruction per gene to distinguish the orthologs from the paralogous, and BUSCOs uses by definition only single-copy orthologs, SiLiX clusters might contain orthologous and paralogous genes (hence a possible explanation for the reduced number of clusters compared with OrthoFinder).

BUSCOs: Finally, we generated a third dataset of single-copy orthologous genes using BUSCOs results we used to assess transcriptome completeness on which we removed potential contaminants. A total of 1,869 clusters containing sequences for at least 80% of the species were recovered (out of 2,510 BUSCOs in OrthoDB v10 Hemiptera).

Once the clusters of orthologous genes were defined for each method, we applied a custom PERL script to select a single sequence per species as multiple sequences might be present for a given species in the cluster. First, we selected for each species the transcript with cumulative best scores against the transcripts of other species in an all-against-all BLASTP. Manual checking of the SiLiX clusters results showed the existence of some chimeric clusters (containing two or more different genes) due to long conserved domains. To resolve this, in a second step we sorted the transcripts by value, selected the transcripts with highest and lowest cumulative score to start and assigned the rest of transcripts to one or the other based on their hit *P*-values. This effectively divided the transcripts in two lists. Third, we selected the longest list and repeated the same list division process again. Finally, for each missing species in the longest list we selected the best hit transcript in the cluster. This method allowed us to select the more conserved transcripts for each species and manual check showed that it effectively solved the problems of chimeric SiLiX clusters.

Manual checking of clusters alignments also showed the presence of gaps that might be filled with other transcripts of the cluster. This situation might be due either a real gene split in two, or most probably, due to a de novo transcriptome assembly artifact. As trying to fill the gaps or merge transcripts might increase the alignment size across species, we decided to remain cautious and avoid possible propagation of errors from a chimeric transcript in one species (i.e., a chimeric transcript in one species will appear as a gap in all the remaining species and might eventually lead to chimeric constructions in all the other species).

### Alignment, Selection of Conserved Blocks and Check for Model Violation Partitions

Prior to phylogeny reconstruction each of the transcript clusters from OrthoFinder, SiLiX, and BUSCOs were aligned using MAFFT (Katoh and Standley 2013), and we used GBLOCKS (Castresana 2000) to retrieve conserved codon regions (options: -t = c -b5 = h). We then concatenated per gene the blocks covering >50% of the gene size and at least 100 nucleotides. This resulted in 1,645, 1,923 and 1,329 gene blocks for OrthoFinder, SiLiX, and BUSCOs, respectively. To avoid partitions that can violate common assumptions in most substitution models, we tested each cluster to verify if nucleotic data was stationary, reversible, and homogeneous (SRH) (Naser-Khdour, et al. 2019). A total of 686, 851, and 528 gene clusters passed the SRH test at position 1 and 2 in OrthoFinder, SiLiX, and BUSCOs, respectively. Third codon position was discarded as it only fulfilled SRH condition in up to three clusters. By not considering position 3 we also avoided possible substitution saturation problems in our analyses. Finally, for all genes which position 1 and 2 passed the SRH test, we generated an aminoacid dataset from their conserved codon regions. Nucleotide dataset contained 261,936, 374,586 and 203,920 positions while aminoacid dataset contained 130,968, 187,293 and 101,960 positions for OrthoFinder, SiLiX, and BUSCOs, respectively. An overview figure of the workflow steps can be found in supplementary fig. S11, Supplementary Material online.

### Phylogeny Reconstruction

To reconstruct a phylogeny, we used two approaches: Maximum-Likelihood (ML) with IQ-TREE (Nguyen, et al. 2015) and Maximum-Parsimony (MP) with TNT (Giribet 2005). In both cases, we used nucleotides (position 1 + 2) and aminoacids matrix resulting from OrthoFinder, SiLiX, and BUSCOs clusters.

Maximum-Likelihood approach: To identify the best partitioning schemes and determine the best amino acid substitution model for each partition we used ModelFinder within IQ-TREE (Nguyen, et al. 2015; Kalyaanamoorthy, et al. 2017) (option: -m TESTMERGE) and an edge unlinked model (option: -sp) (Chernomor, et al. 2016). To save time we restricted the search to substitution models: LG, JTT, JTTDCMUT, MTZOA, DAYHOFF, and WAG (option: -mset LG, JTT, JTTDCMUT, MTZOA, DAYHOFF, WAG) and limited the computation to the top 10% partition schemes (option: -rcluster 10). We then selected the models that minimize *Bayesian information criterion* (BIC) score and launch a new run to reconstruct a tree using the best partitions schemes found (option: -m MFP), resampling partitions and then resampling sites within resampled partitions (Gadagkar, et al. 2005; Seo, et al. 2005) (option: -bsam GENESITE). To provide robustness estimates for the resulting maximum likelihood tree we generated 1,000 bootstrap replicates (option: -bb 1,000) using ultrafast bootstrap (UFBoot) approximation (Minh, et al. 2013). To reduce the risk of overestimated branch support, we used nearest neighbor interchange (NNI) option (-bnni). Finally, to asses branch supports we used single branch tests in combination with UFBoot (options: -alrt 1,000) (supplementary figs. S12–S14, Supplementary Material online). We used same parameters to reconstruct nucleotides trees from amino acids merged partitions (supplementary figs. S15–S17, Supplementary Material online).

Maximum-Parsimony approach: To reconstruct the trees using maximum-parsimony, we used TNT (Giribet 2005) on amino acid sequences with 100 replications using TBR branch swapping (option: mult = replic 100 tbr) to get branch lengths, and resampling 1,000 times the matrix to calculate group support (option: resample replications 1,000) (supplementary figs. S18–S20, Supplementary Material online).

Once all reconstructions done, we used TreeShrink package (Mai and Mirarab 2018) to detect outlier long branches in all our reconstructed trees. A single long branch corresponding to *Neogerris hesione* was detected, although not in all reconstructed trees. Additionally, although *Velia caprai* was consistently branching sister to the rest of (“Vellidae” + Gerridae), we wondered if its position could explain the early divergence found between those two groups and the (Hydrometridae + Hebridae + Mesoveliidae). To test this hypothesis and discard any possible artifact of those two species on tree reconstruction, we removed those two species from OrthoFinder data and repeated the tree reconstruction using IQ-TREE for aminoacid and nucleotide sequences as described previously (supplementary figs. S21 and S22, Supplementary Material online). Finally, we also built complementary trees with IQ-TREE for BUSCOs data with no partition merging (option: -bb 1,000 -bsam GENESITE) to test robustness of the merging results (supplementary figs. S23 and S24, Supplementary Material online). Overall, all tree reconstruction approaches find the same relationships between the Gerromorpha families and subfamilies regardless of the dataset and method used (supplementary figs. S1 to S4, Supplementary Material online). However, branch supports, and more particularly UFBOOT values, are worse in trees reconstructed with SiLiX datasets which might indicate the presence of paralogous genes hindering the signal, as discussed in orthologous genes cluster section.

### Likelihood Mapping Analyses

A final additional likelihood mapping analysis was performed to confirm early split of (“Hydrometridae”+Hebridae + Mesoveliidae) and (“Veliidae”+Gerridae) as well as sister position of “Hydrometridae” to (Hebridae + Mesoveliidae). To do so, we used IQ-TREE (option: -n 0 -lmap 4,000 -m TEST -mset LG, JTT, JTTDCMUT, MTZOA, DAYHOFF, WAG) to test possible topologies between: Outgroups, Hydrometrinae, (Hebridae + Mesoveliidae + Heterocleptinae) and (“Veliidae”+Gerridae) (supplementary fig. S25*A* and *B*, Supplementary Material online). Similar to phylogenetic reconstruction we tested possible topologies with and without *Velia caprai* with similar results (supplementary fig. S25*C* and *D*, Supplementary Material online, respectively). Results show that our reconstructed tree topology is well supported, but almost 25% of the quartets support alternative topology with (Hebridae + Mesoveliidae + Heterocleptinae) being sister to (Hydrometrinae + [“Veliidae”+Gerridae]) which reinforces the importance of using large datasets when possible.

### Fossil Calibration

Estimated age of seven fossils of extinct Gerromorpha species, two extinct Nepomorpha species and two extinct Heteroptera were used to calibrate divergence time at six points of our phylogenetic tree (supplementary material S3, Supplementary Material online). To date the phylogeny, we use MCMC implementation in BEAST2 (Bouckaert, et al. 2019) using accompanying GUI BEAUTi to build the launch file. As starting tree we used IQ-TREE phylogenetic trees from OrthoFinder nucleotide data. To increase the site coverage, we retained only positions present in 98% taxa which accounted for 45,672 nucleotides. We fixed the topology by setting “Wide-exchange”, “Narrow-exchange”, “Wilson-Balding”, and “Subree-slide” weight to zero and ran five independent runs of 200,000,000 generations using a Birth Death Model sampling every 1,000 generations. We analyzed the combined results with Tracer (part of BEAST2 package) and all ESS values were higher than 200. We manually combined the five replicates’ trees and a final tree with median ages was constructed using Treeannotator (part of BEAST2 package) with a 25% burnIn. Tree visualization was performed with Figtree 1.4.4 (Rambaut 2018). Root age was estimated at 273.95 Mya (95% CI 252–297.68).

To test if fossil calibration point on *Velia caprai* combined with its position sister to the rest of (‘Vellidae’ + Gerridae) can have an impact on tree dating, in particular if we speculate on a possible reconstruction artifact, we repeated the dating by removing fossil calibration point on *Velia caprai*. We run five new independent runs and combined their results (all ESS higher than 200). Compared to first tree, we obtained with very similar results (i.e., (Mesoveliidae + Hebridae + “Hydrometridae”) and (“Veliidae” + Gerridae) split dated 202.75 Mya and 198.319.1 Mya, respectively, (“Veliinae”+Rhagoveliinae) split dated at 100.41 Mya and 89.53 Mya, respectively, and Microveliinae split from other Gerridae dated 115.18 and 113.3 Mya, respectively. Root age was estimated at 276.08 Mya (95% CI 223.4–306.1 Mya). In this second run, we also removed (Mesoveliinae + Madeoveliinae) calibration point. Indeed, fossil records evidence of both families differ in 50 Mya (see supplementary table S3, Supplementary Material online) and trying to set their divergence at 150 Mya in the initial tree (as suggested by Madeoveliinae fossil) failed to find a proper state to initialize. Therefore, we set the lower boundary distribution to 100 Mya in the first run and used this second run to confirm that split of Madeoveliidae from Mesoveliinae date from 104 Mya (106 Mya in first run) (supplementary fig. S26, Supplementary Material online).

### Ancestral Character State Reconstruction

Reconstruction of ancestral character states was performed in Rstudio using “ape” (Paradis, et al. 2004), “phytools’ (Revell 2012) and “geiger” (Pennell, et al. 2014) packages. First, as suggested by Litsios and Salamin (2012) we computed phylogenetic signal (Pagel lambda) for both phylogram (molecular phylogeny) and chronogram (time-calibrated phylogeny). To do so we use phytools:: phylosig function (method=”lambda”, nsim = 1000). In both cases, we excluded *Negogerris hesione*. Based on the results obtained we retained the phylogram for ancestral character state reconstruction. Second, we used “phytools::make.simmap” function to simulate character maps on the phylogenetic tree. This is an adapted version of “ace” function from “ape” and allows to test three different transition models to reconstruct a discrete trait: equal-rates model (ER), all-rates-different model (ARD) and symmetrical model (SYM). To decide which model fit better our data, we used “geiger::fitDiscrete” function. We checked AICc values and selected the best transition model for each dataset based on a likelihood ratio test. To do so we compared the square distribution with the degrees of freedom to calculate a *P*-value of the likelihood ratio test (Command example: *P*_value_ER_versus_SYM < - pchisq(abs(2*(ER_geiger$opt$lnL-SYM_geiger$opt$lnL)), abs(SYM_geiger$opt$k-ER_geiger$opt$k), lower.tail = FALSE)). Main figures were composed based on best transition model. Additionally, we also computed maximum-parsimony ancestral reconstruction using Rstudio package “castor” (Louca and Doebeli 2018) with transition costs “all_equal” and “exponential”. All model results can be found summarized in supplementary figs. S27–S30, Supplementary Material online. A summary of figs. 3–6 can be found in supplementary fig. S31, Supplementary Material online. For leg length continuous trait, we used “phytools:fastAnc” to estimate ancestral states including variances and 95% confidence intervals (options: vars = TRUE, CI = TRUE).

#### Nymphal RNAi

Double-stranded RNA (dsRNA) was produced for *Ultrabithorax* gene (*Ubx*) as described in (Refki, et al. 2014). T7 PCR fragments were amplified from complementary DNA (cDNA) template using forward and reverse primers both containing the T7 RNA polymerase promoter. The amplified fragments were purified using the QIAquick PCR purification kit (Qiagen, France) and used as a template to synthesize the dsRNA as described in Refki, et al. (2014). The synthetized dsRNA was then purified using a RNeasy purification kit (Qiagen) and eluted in Spradling injection buffer (Rubin and Spradling 1982) in a 2–3 μg/μl concentration. For primer information, see Refki, et al. (2014). Nymphal injections were performed in parallel in nymphs with either *Ubx* dsRNA or injection buffer as negative controls. Nymphs were reared in water tanks and fed with crickets until they developed into adults. We then compared adults obtained from *Ubx* dsRNA injection to those obtained from injection of buffer injection alone.

## Supplementary Material

msac229_Supplementary_Data

## Data Availability

BioProject ID: PRJNA774202.

## Supplementary Material

Scripts used to define the conserved blocks can be found here: https://gitlab.com/davidarmisen/transcriptomic-based-phylogeny-in-gerromorpha. A general overview of the scripts pipeline can be found at supplementary fig. S11, Supplementary Material online. A more detailed description of each script function can be found in “master-script.pl” file.

Aligned conserved blocks from OrthoFinder, SiLiX, and BUSCOs used for phylogeny reconstruction can be found in the same site.

Additionally, a BLAST web page including transcriptome sequences of the ninety-two Gerromorpha, two Notonectidae, and one Pyrrhocoridae (supplementary table S1, Supplementary Material online) we newly sequenced, plus *Limnoporus dissortis* and *Gerris buenoi*, can be found at: https://igfl.ens-lyon.fr/equipes/a.-khila-developmental-genomics-and-evolution/blast.
