# Supplementary figures and images for: Transcriptome-based Phylogeny of the Semi-aquatic Bugs (Hemiptera: Heteroptera: Gerromorpha) Reveals Patterns of Lineage Expansion in a Series of New Adaptive Zones
Source: Mol Biol Evol. 2022 Oct 21;39(11):msac229. doi: 10.1093/molbev/msac229 (PMC9641996; doi:10.1093/molbev/msac229)

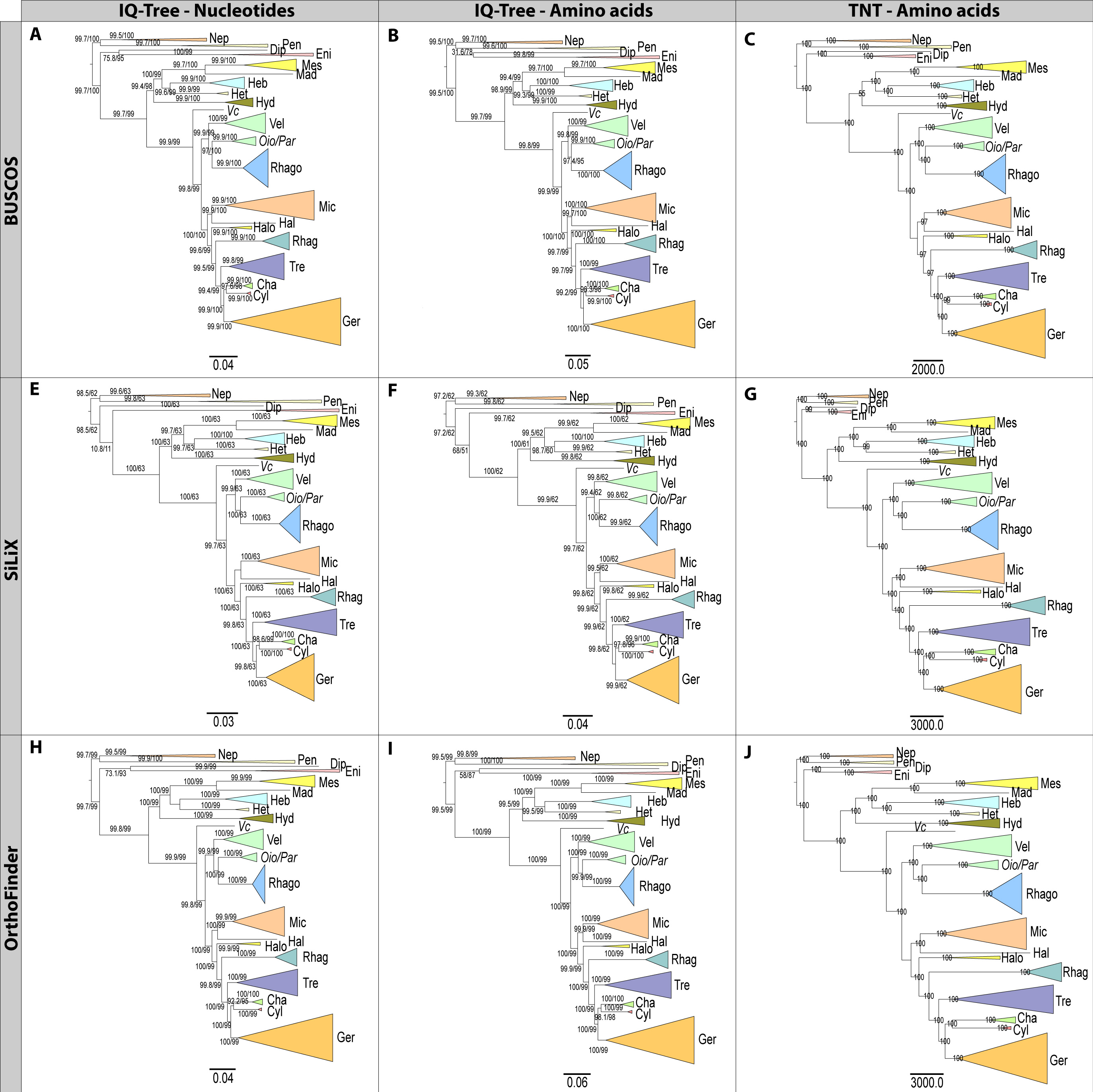

Supplement: msac229_Supplementary_Data [file msac229_supplementary_data.zip › Supplementary_Figure_1.jpg]

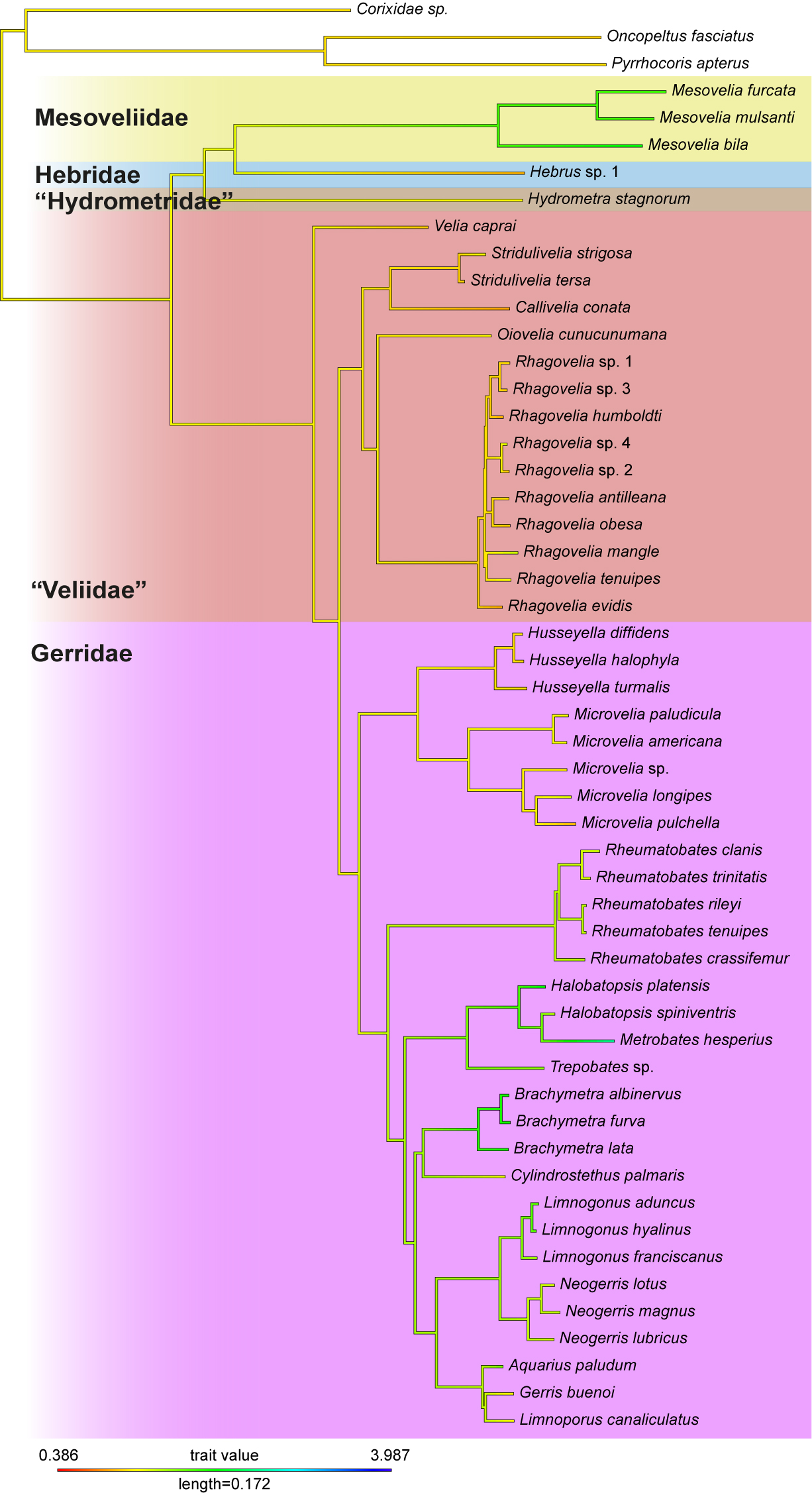

Supplement: msac229_Supplementary_Data [file msac229_supplementary_data.zip › Supplementary_Figure_10.jpg]

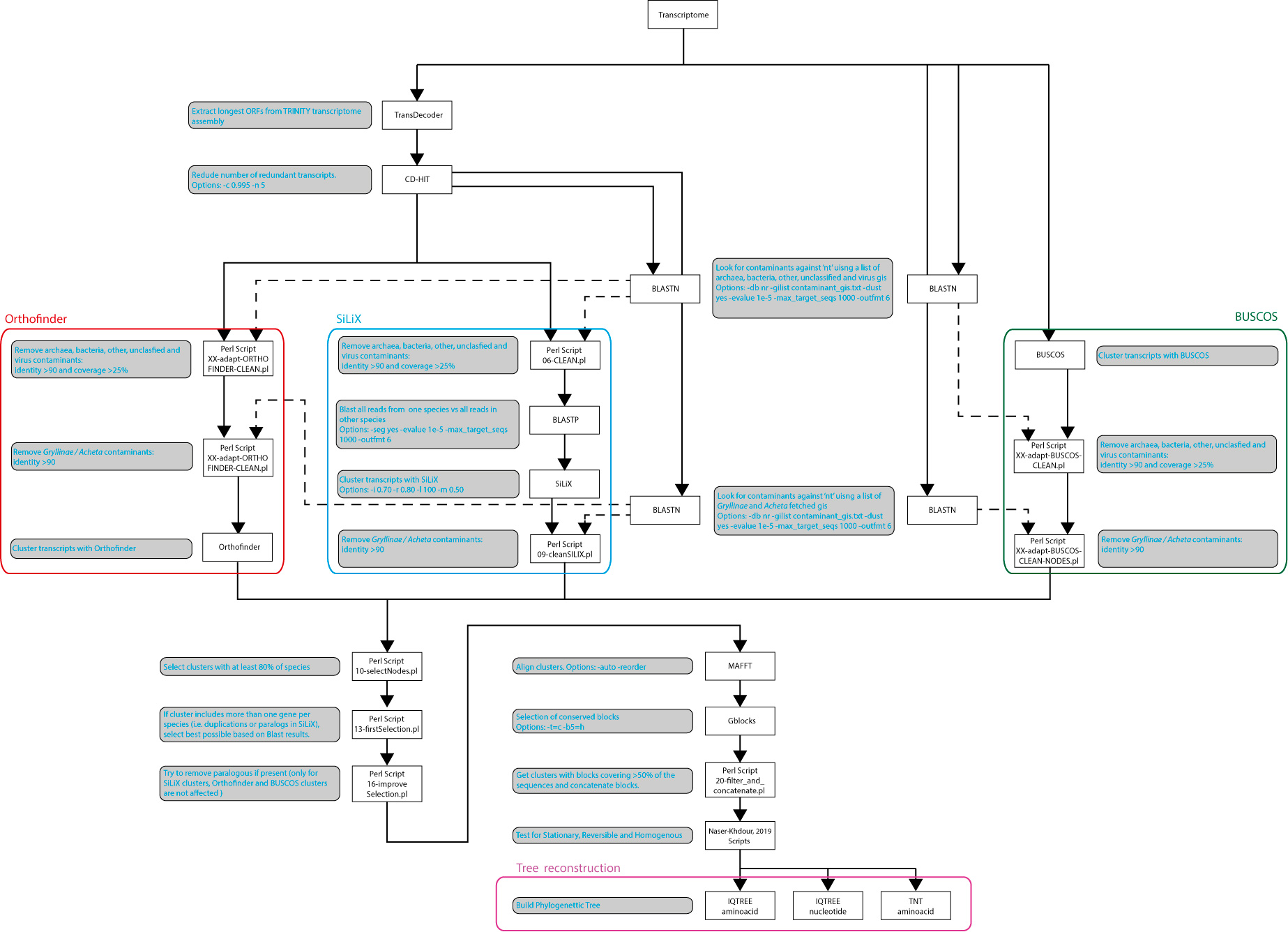

Supplement: msac229_Supplementary_Data [file msac229_supplementary_data.zip › Supplementary_Figure_11.jpg]

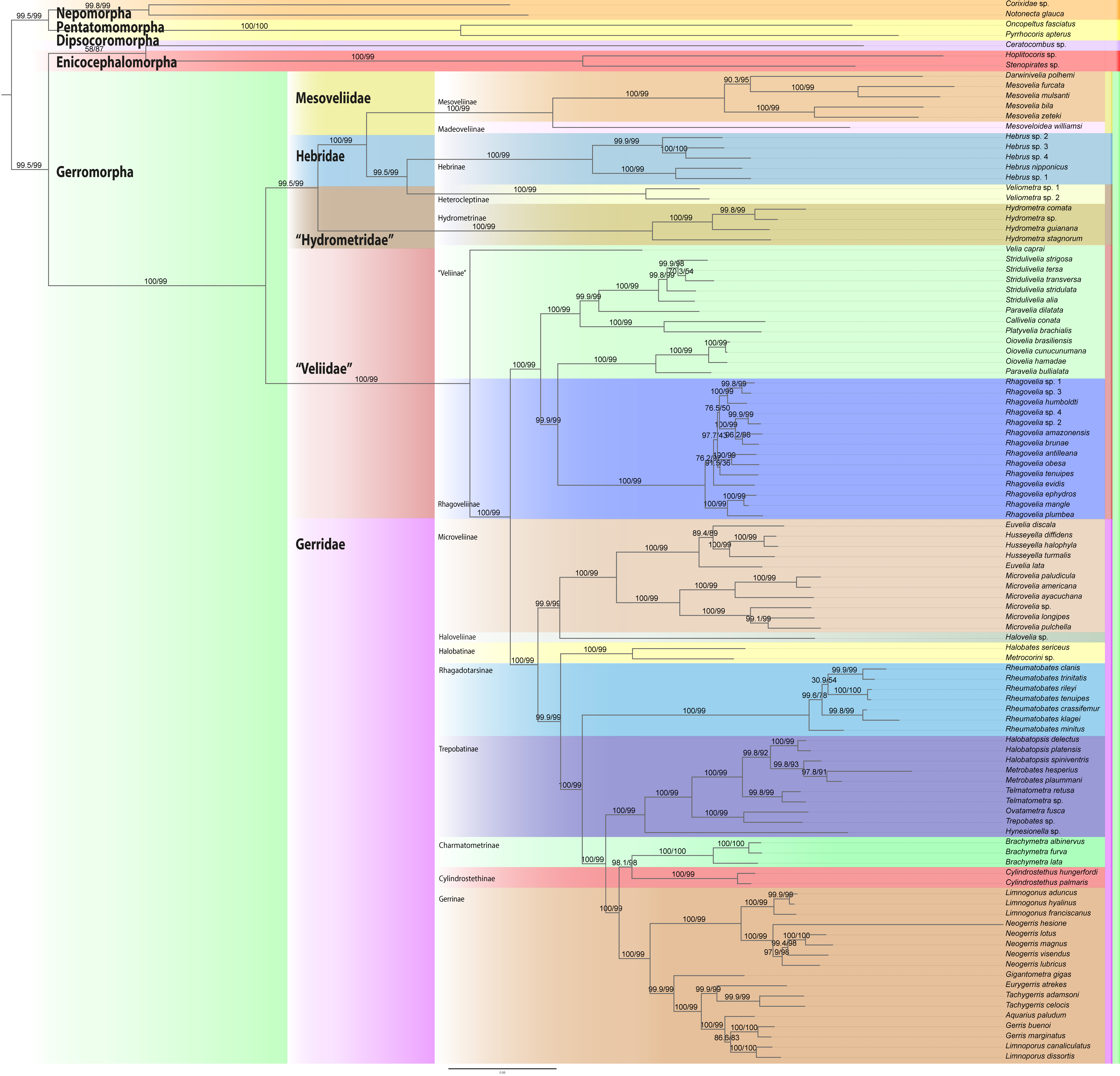

Supplement: msac229_Supplementary_Data [file msac229_supplementary_data.zip › Supplementary_Figure_12.jpg]

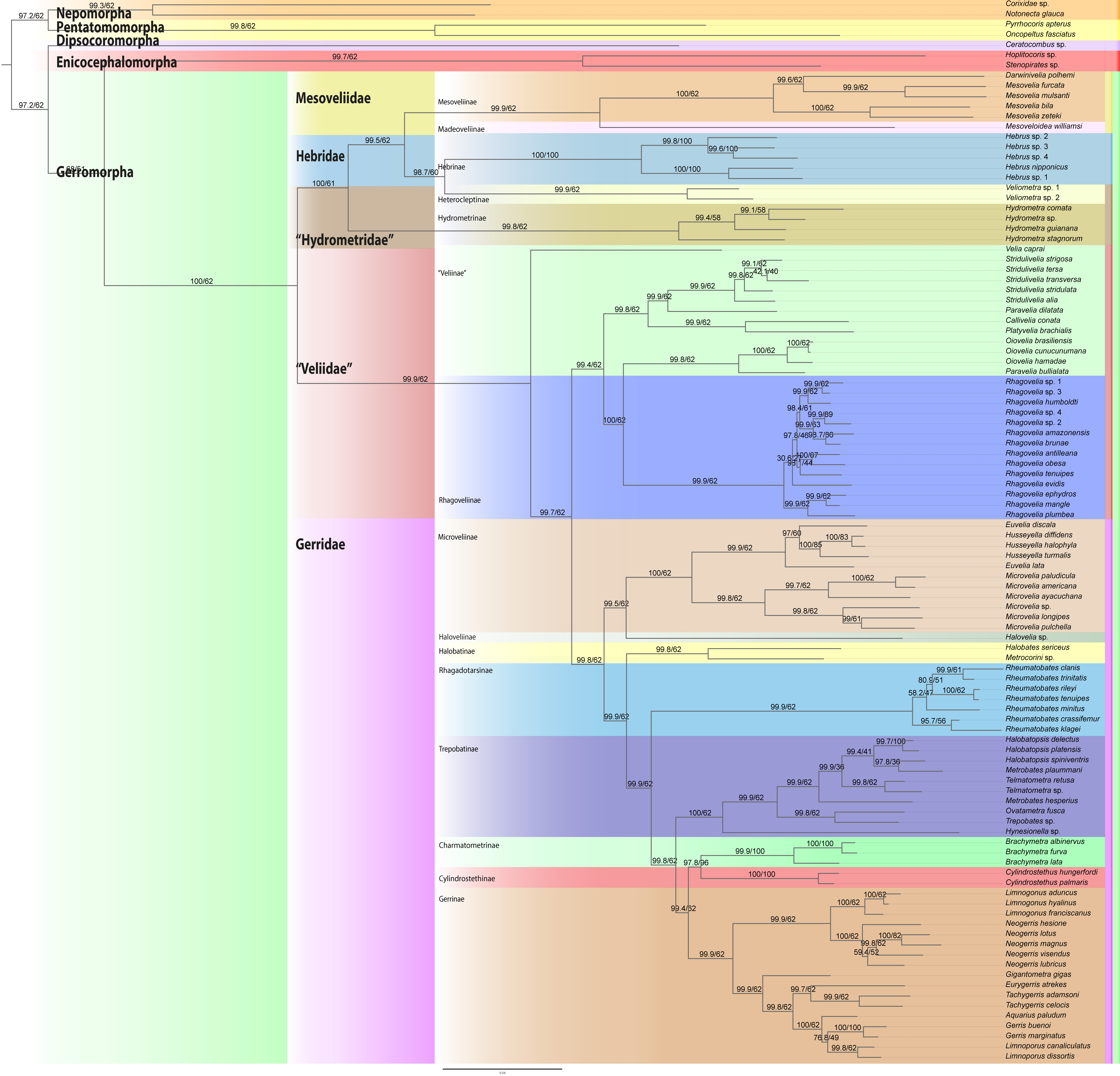

Supplement: msac229_Supplementary_Data [file msac229_supplementary_data.zip › Supplementary_Figure_13.jpg]

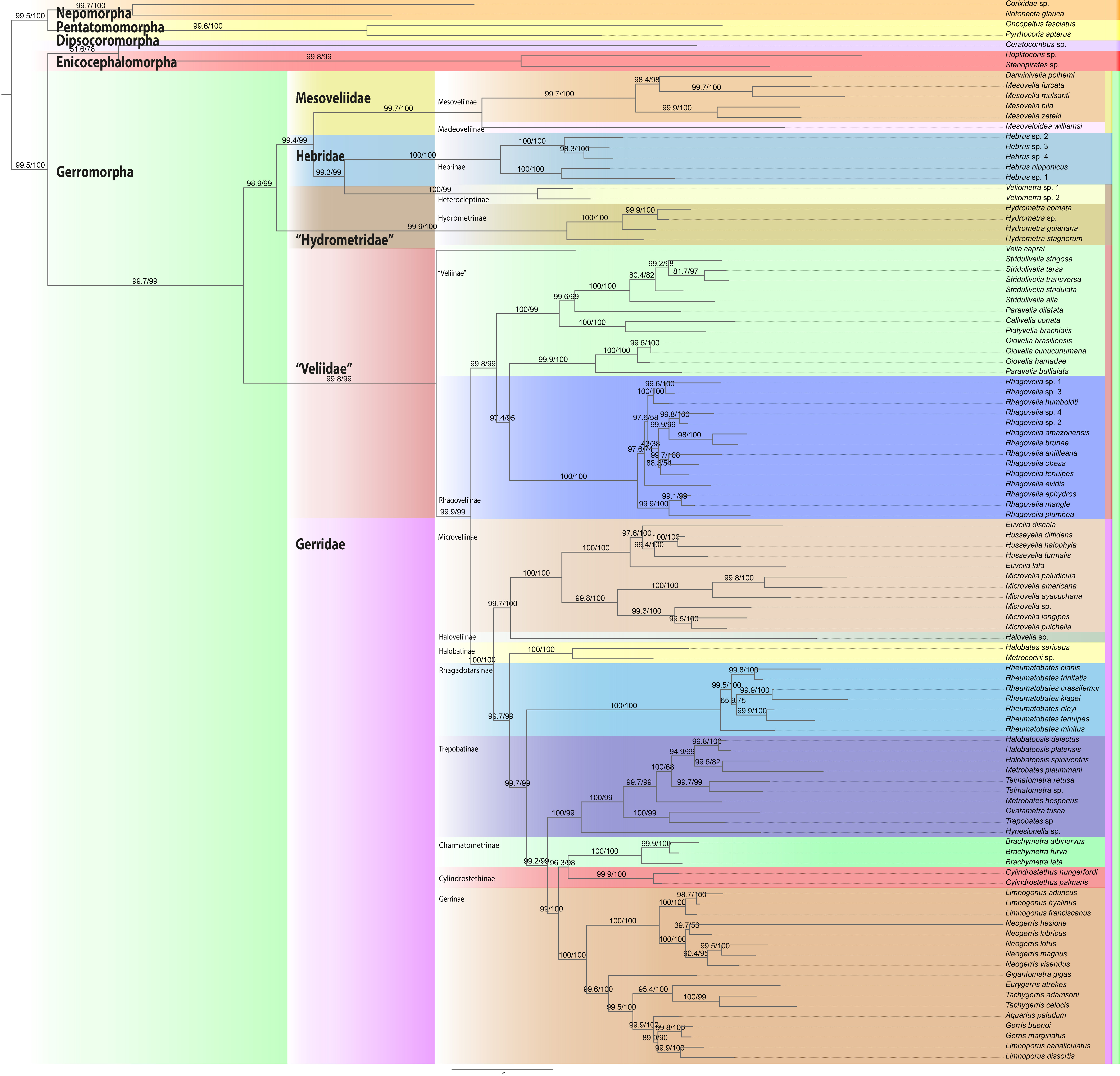

Supplement: msac229_Supplementary_Data [file msac229_supplementary_data.zip › Supplementary_Figure_14.jpg]

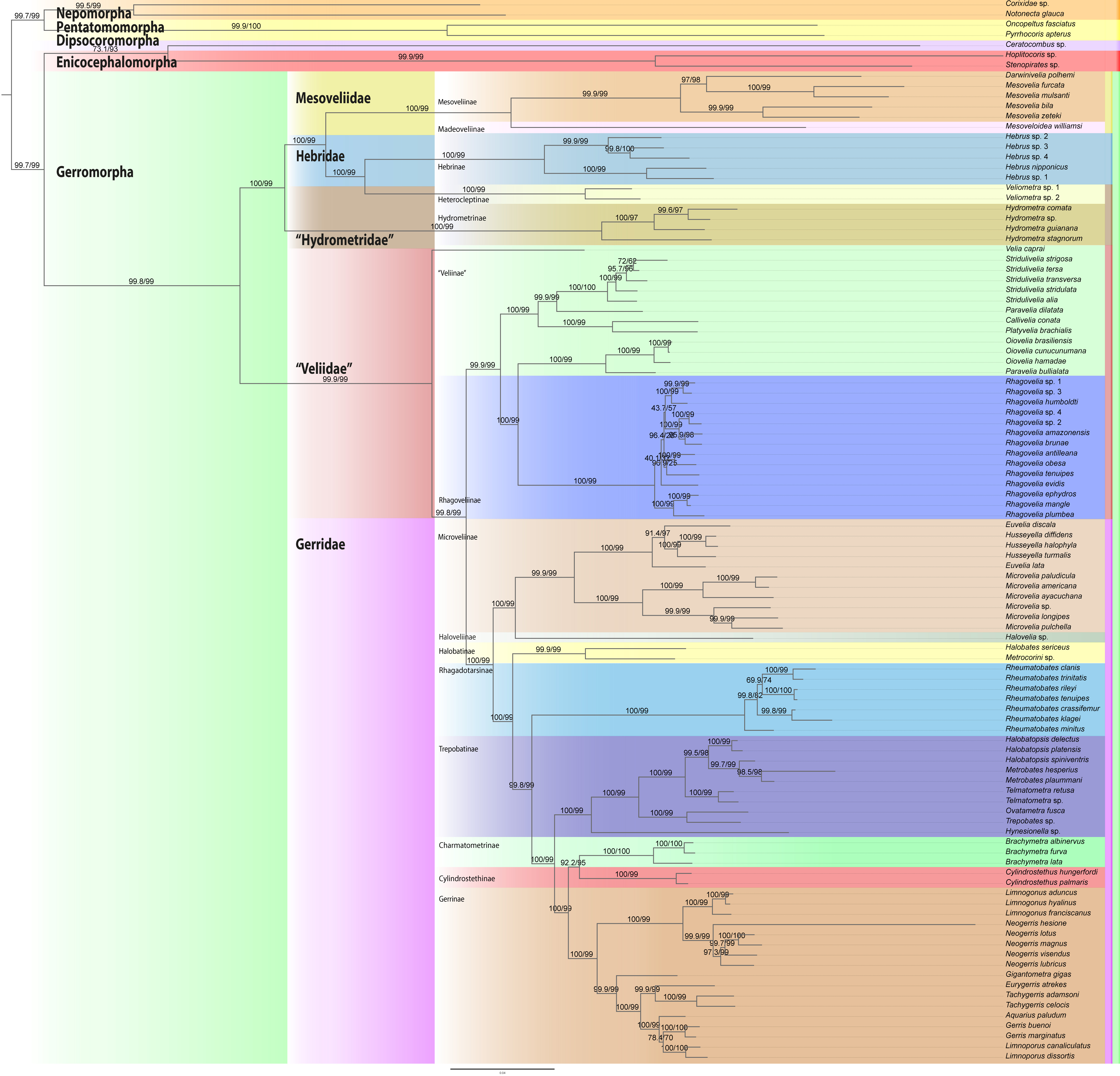

Supplement: msac229_Supplementary_Data [file msac229_supplementary_data.zip › Supplementary_Figure_15.jpg]

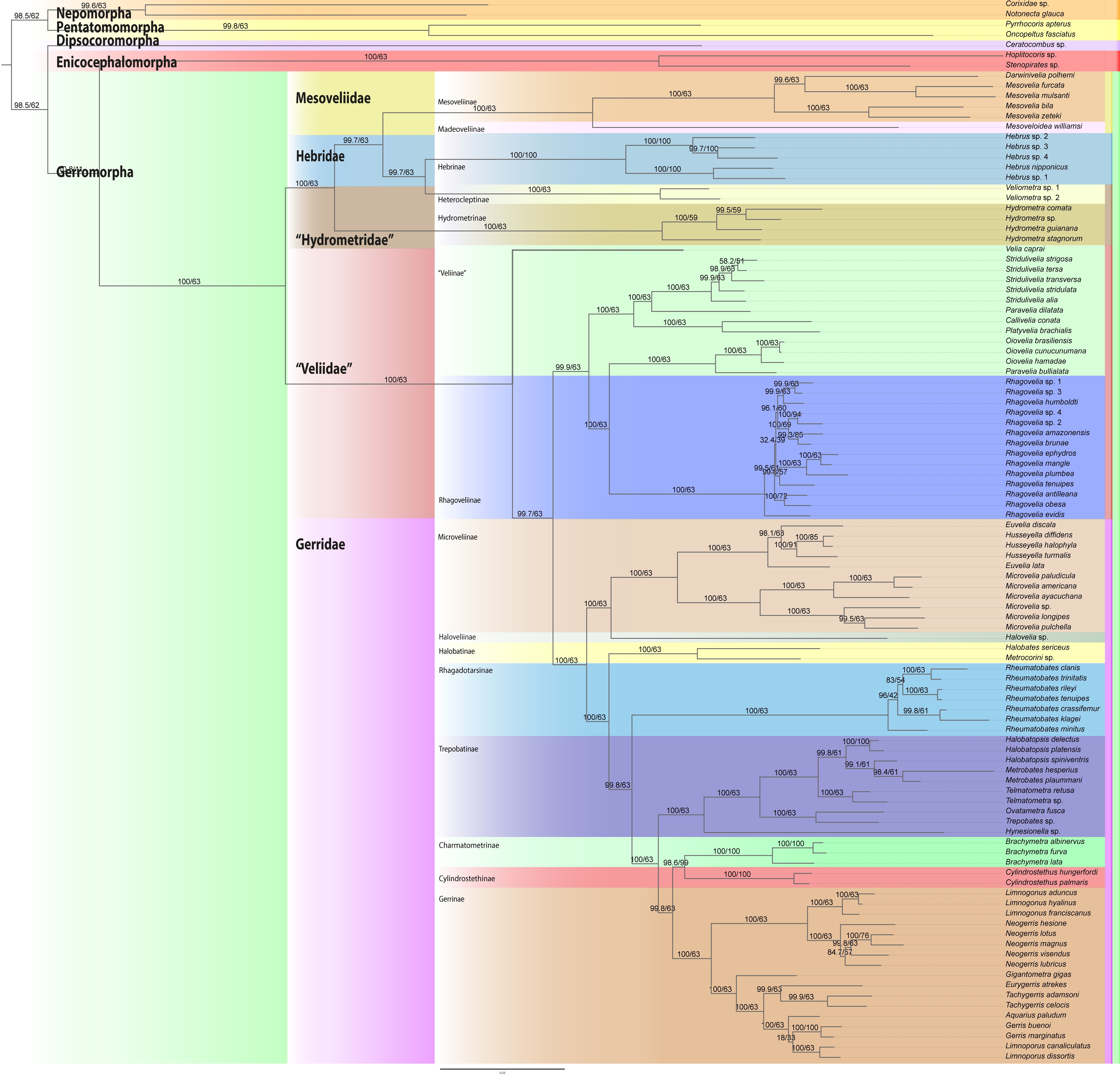

Supplement: msac229_Supplementary_Data [file msac229_supplementary_data.zip › Supplementary_Figure_16.jpg]

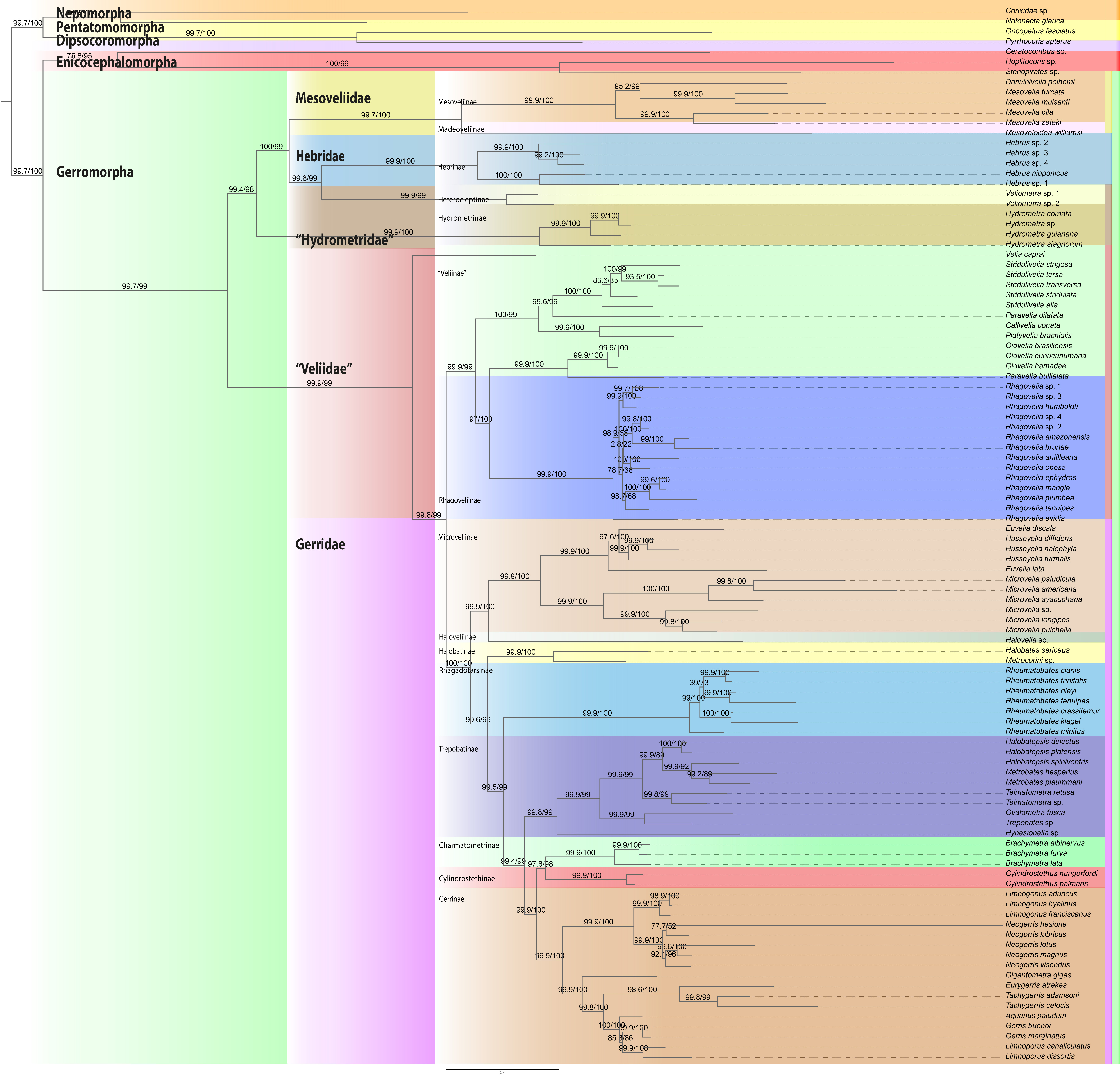

Supplement: msac229_Supplementary_Data [file msac229_supplementary_data.zip › Supplementary_Figure_17.jpg]

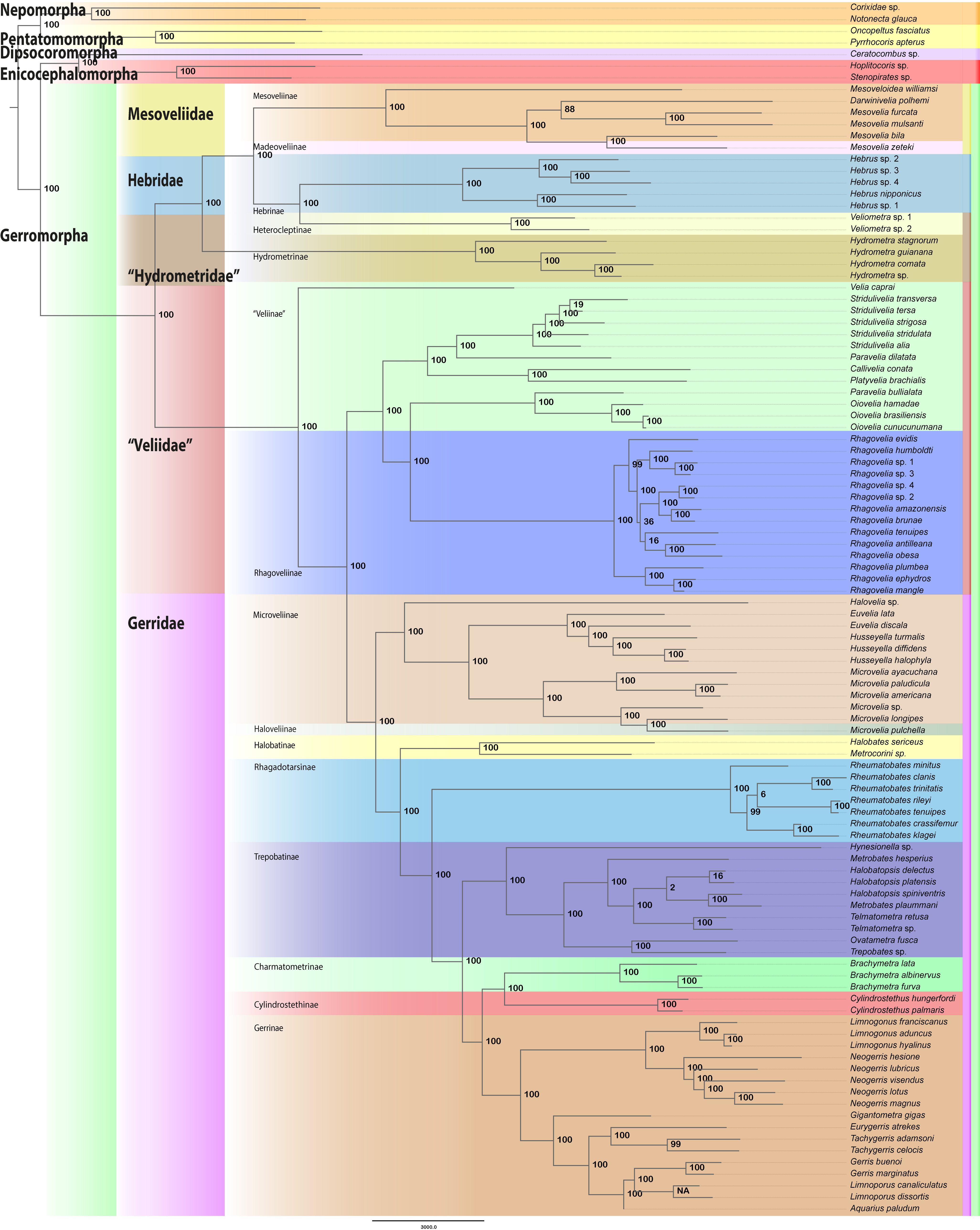

Supplement: msac229_Supplementary_Data [file msac229_supplementary_data.zip › Supplementary_Figure_18.jpg]

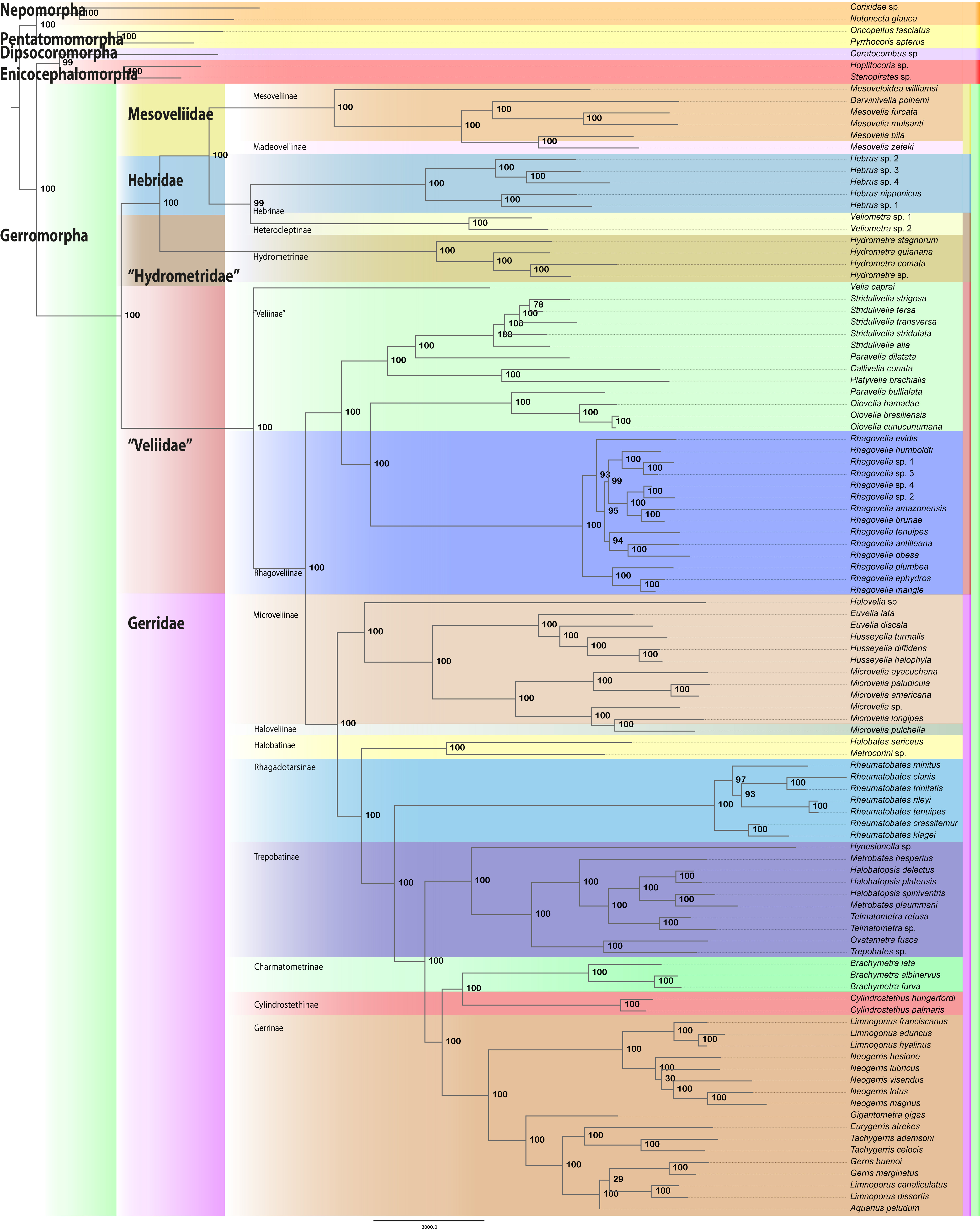

Supplement: msac229_Supplementary_Data [file msac229_supplementary_data.zip › Supplementary_Figure_19.jpg]

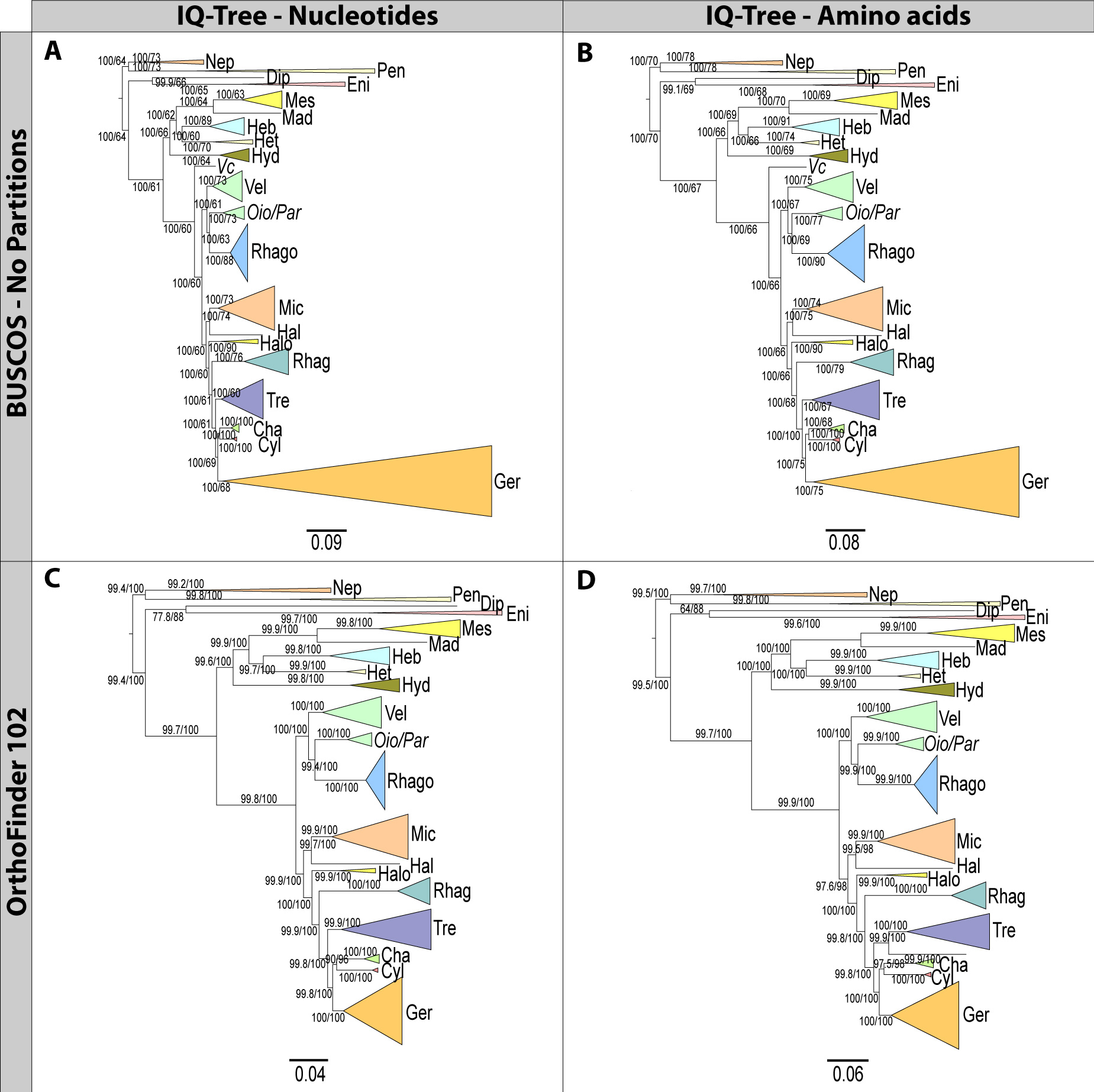

Supplement: msac229_Supplementary_Data [file msac229_supplementary_data.zip › Supplementary_Figure_2.jpg]

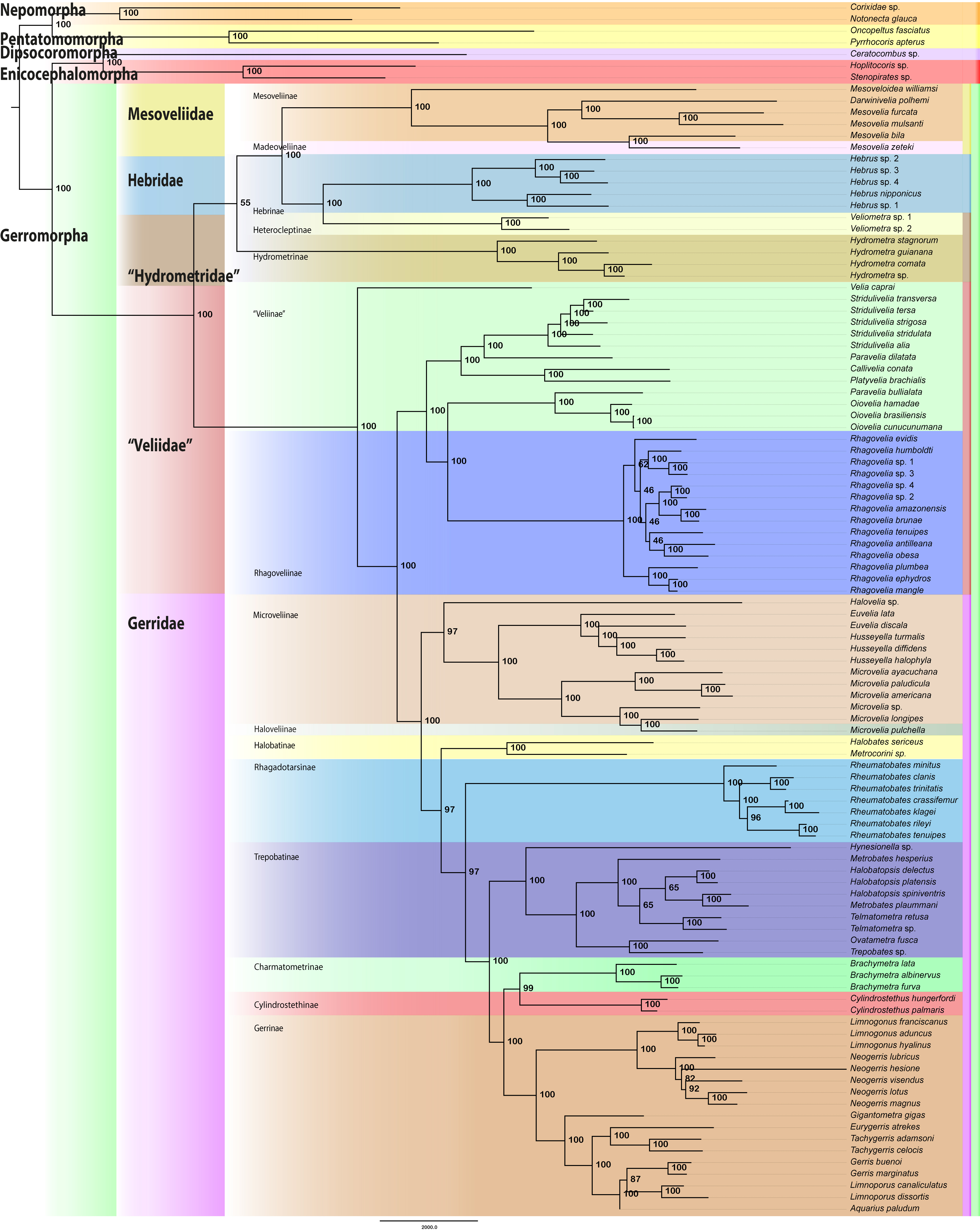

Supplement: msac229_Supplementary_Data [file msac229_supplementary_data.zip › Supplementary_Figure_20.jpg]

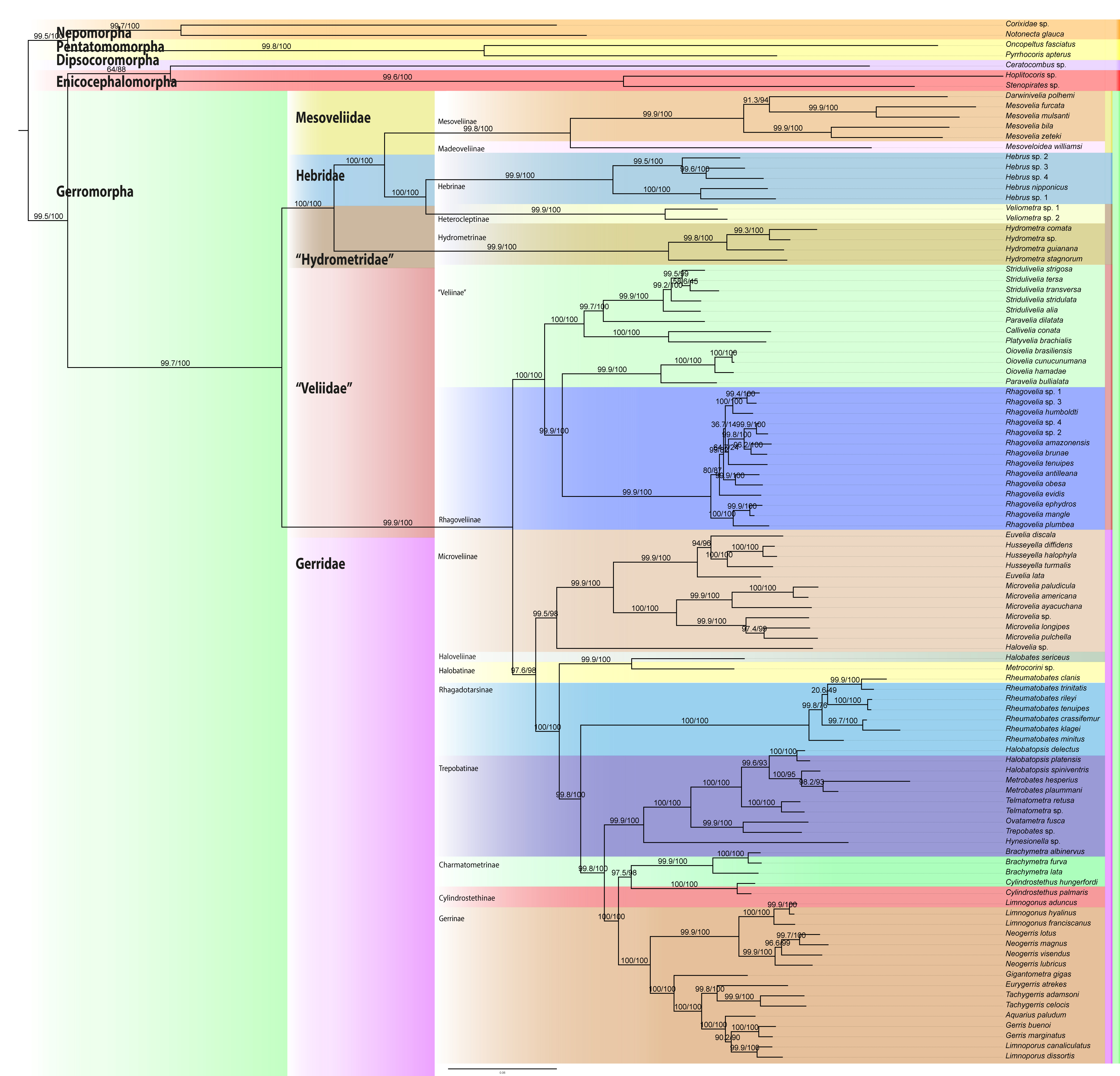

Supplement: msac229_Supplementary_Data [file msac229_supplementary_data.zip › Supplementary_Figure_21.jpg]

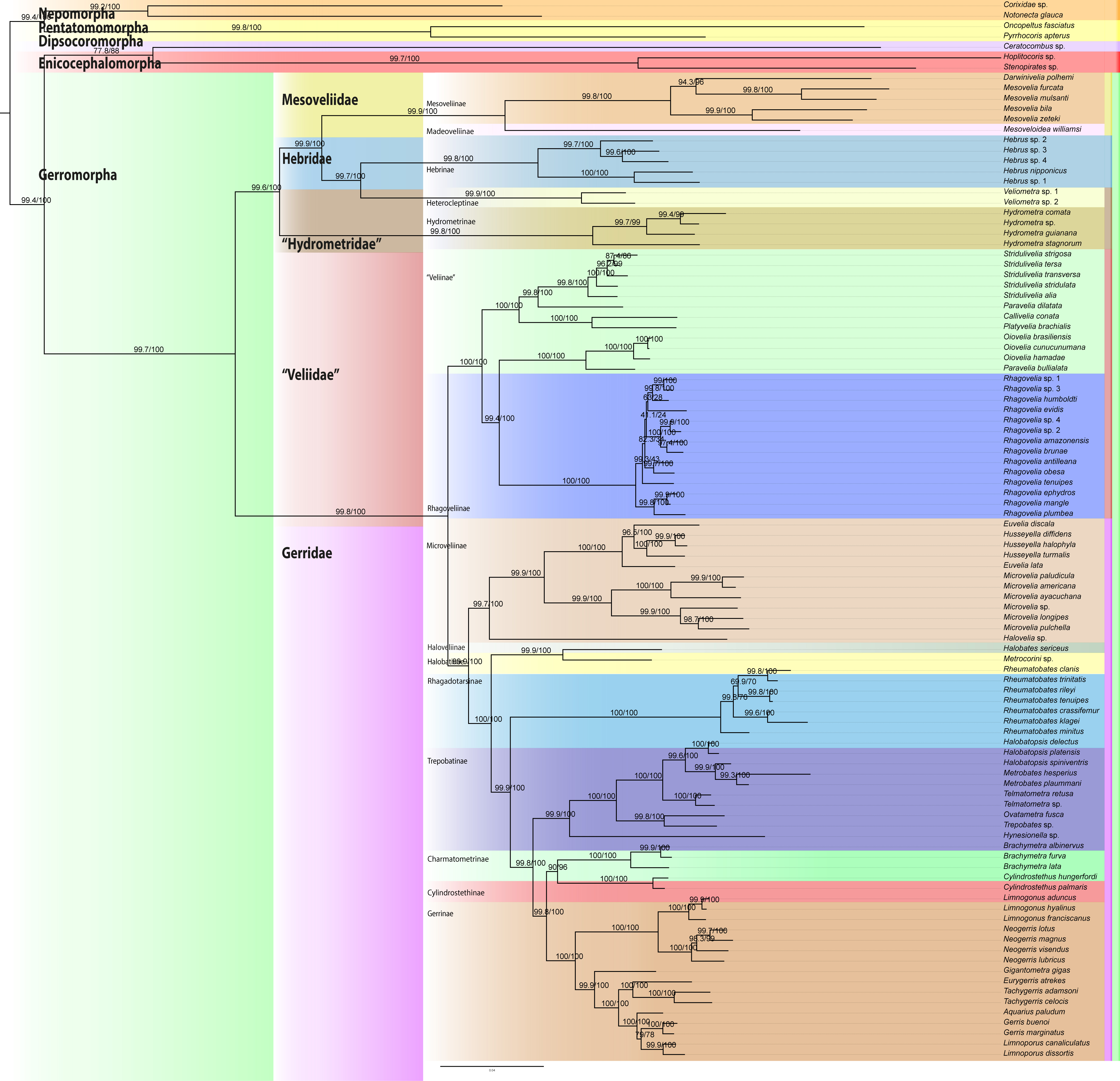

Supplement: msac229_Supplementary_Data [file msac229_supplementary_data.zip › Supplementary_Figure_22.jpg]

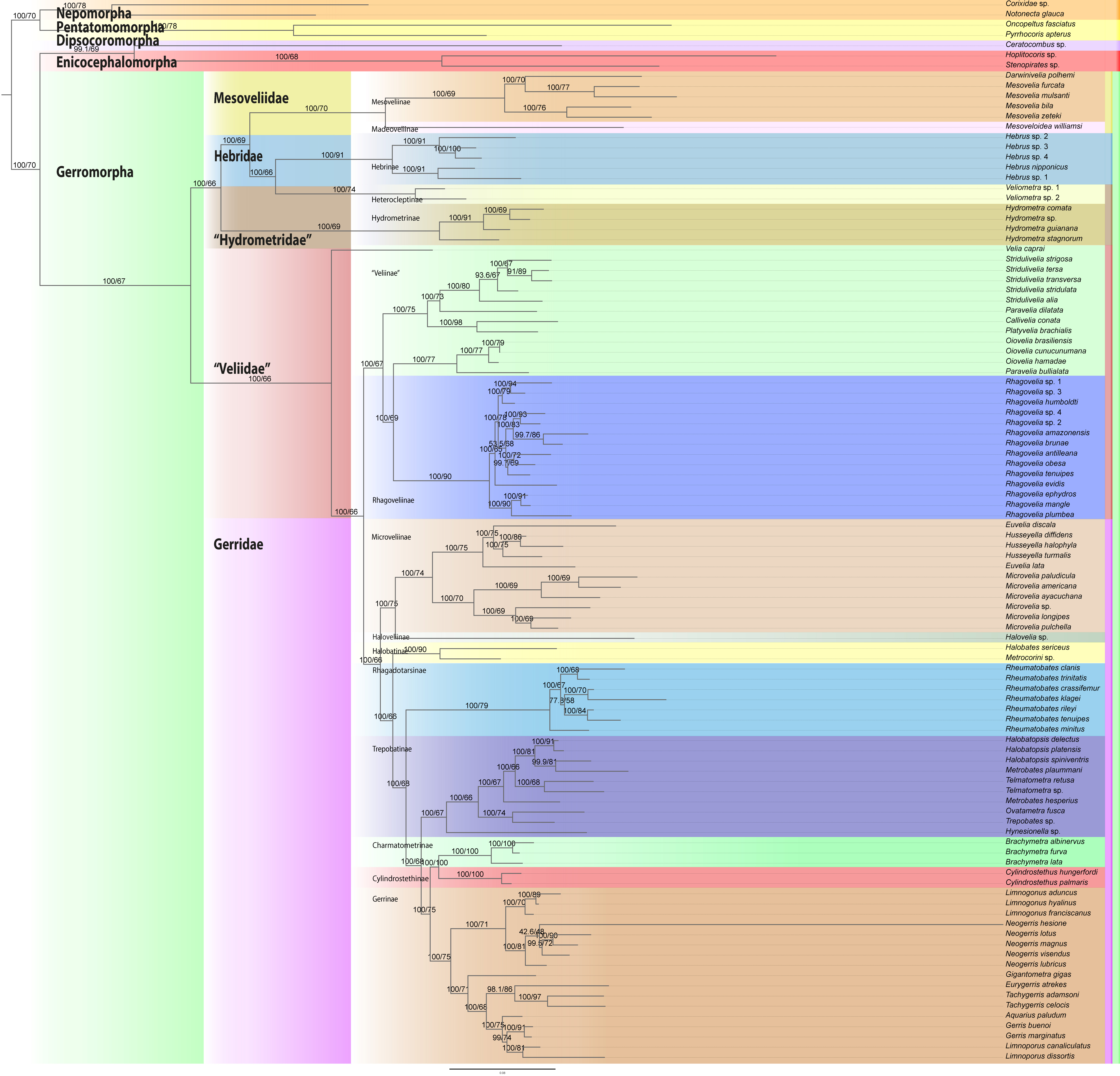

Supplement: msac229_Supplementary_Data [file msac229_supplementary_data.zip › Supplementary_Figure_23.jpg]

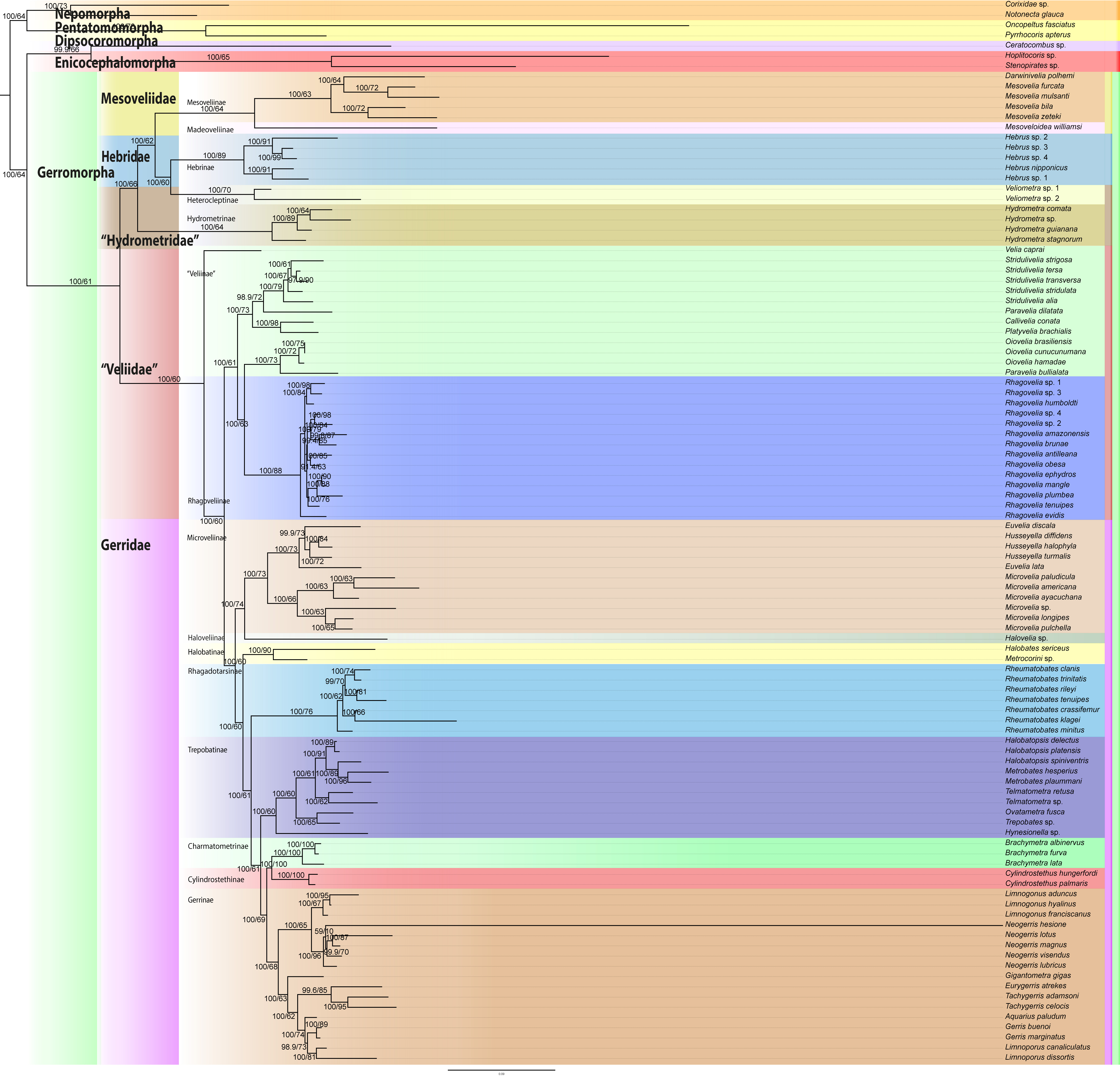

Supplement: msac229_Supplementary_Data [file msac229_supplementary_data.zip › Supplementary_Figure_24.jpg]

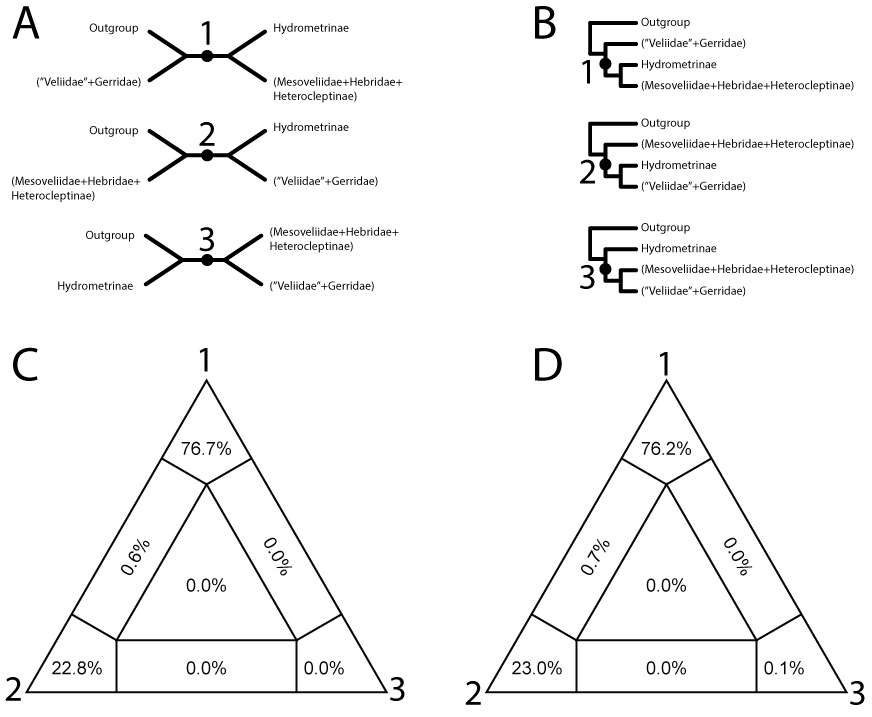

Supplement: msac229_Supplementary_Data [file msac229_supplementary_data.zip › Supplementary_Figure_25.jpg]

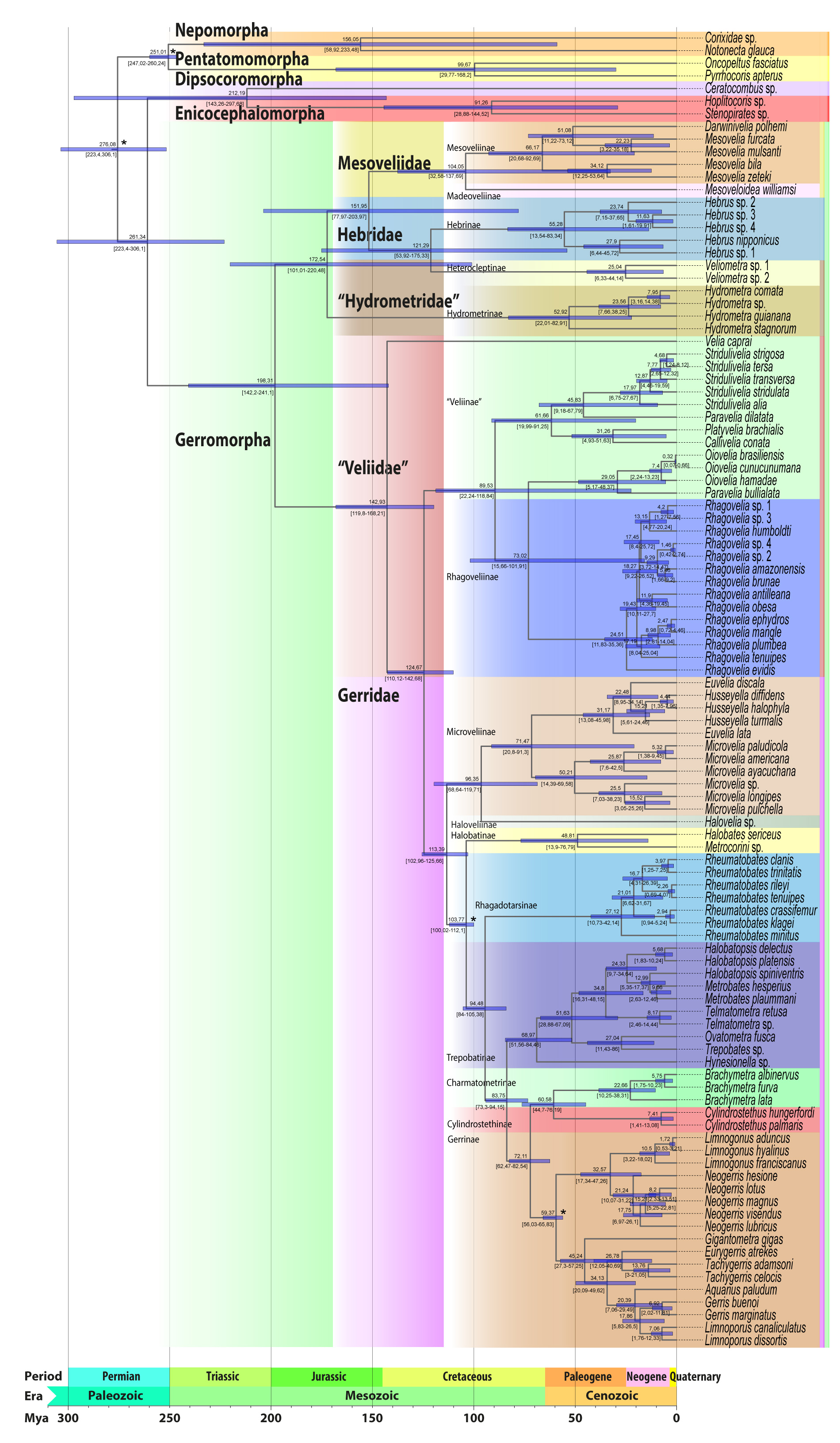

Supplement: msac229_Supplementary_Data [file msac229_supplementary_data.zip › Supplementary_Figure_26.jpg]

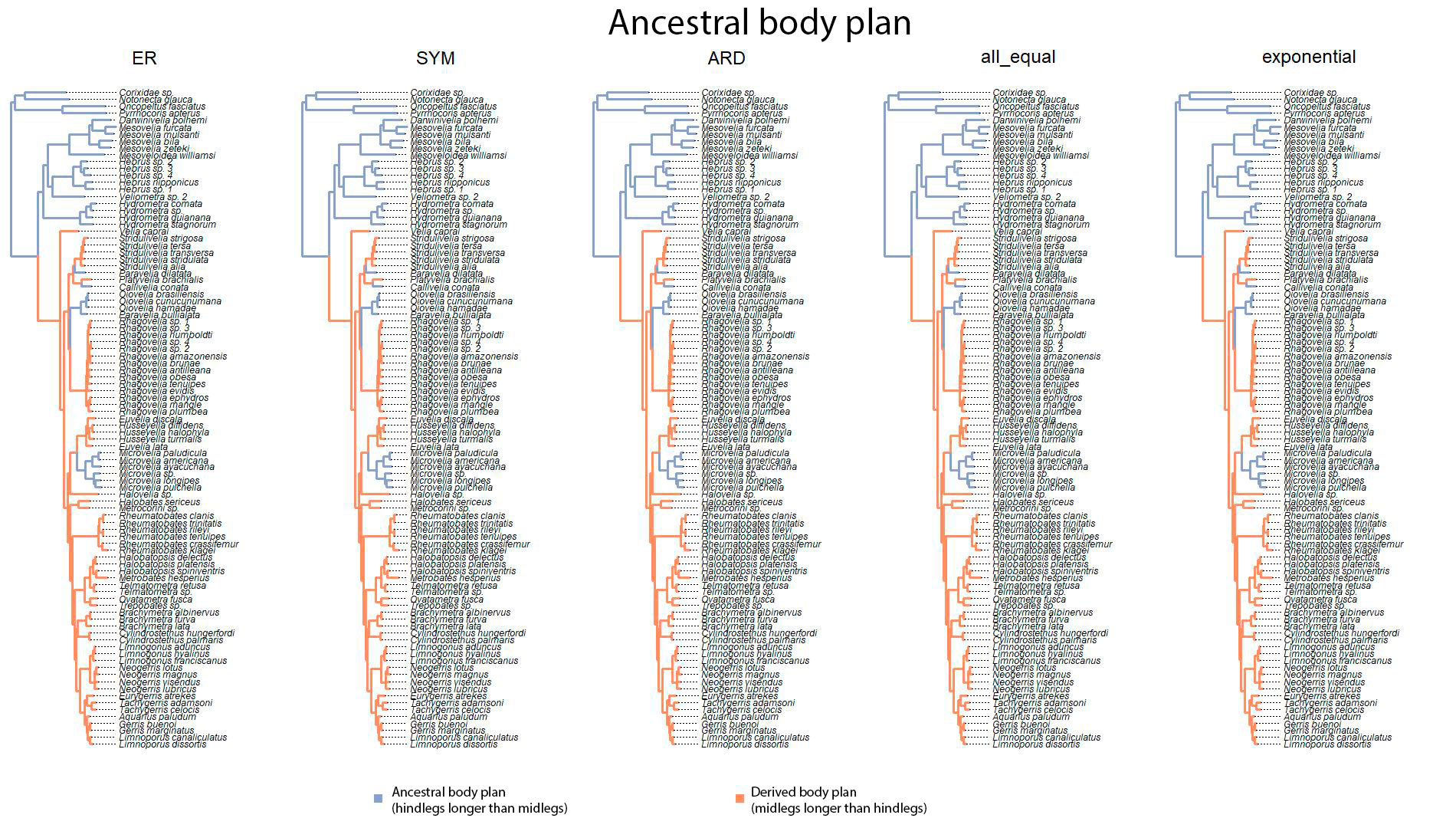

Supplement: msac229_Supplementary_Data [file msac229_supplementary_data.zip › Supplementary_Figure_27.jpg]

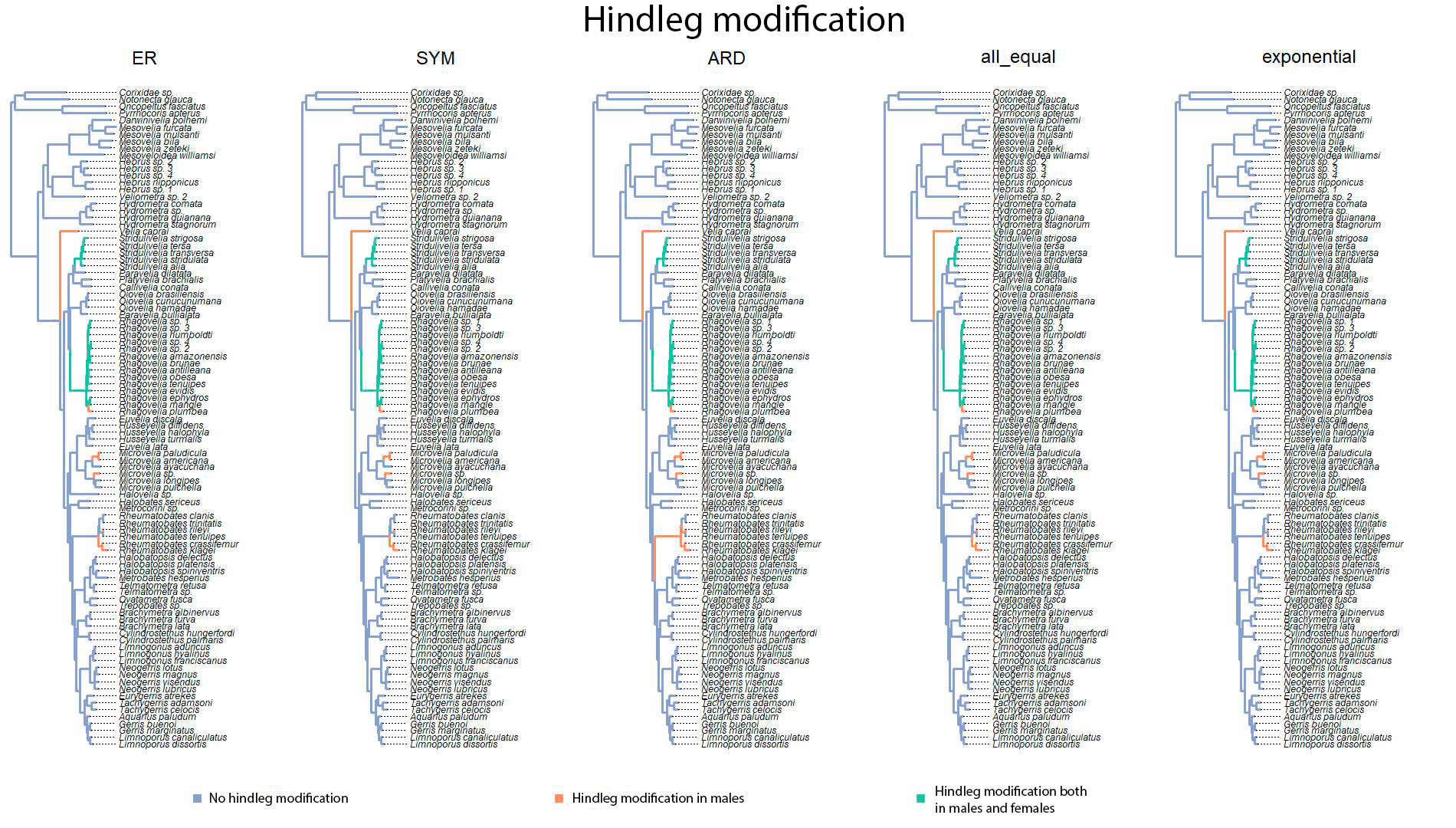

Supplement: msac229_Supplementary_Data [file msac229_supplementary_data.zip › Supplementary_Figure_28.jpg]

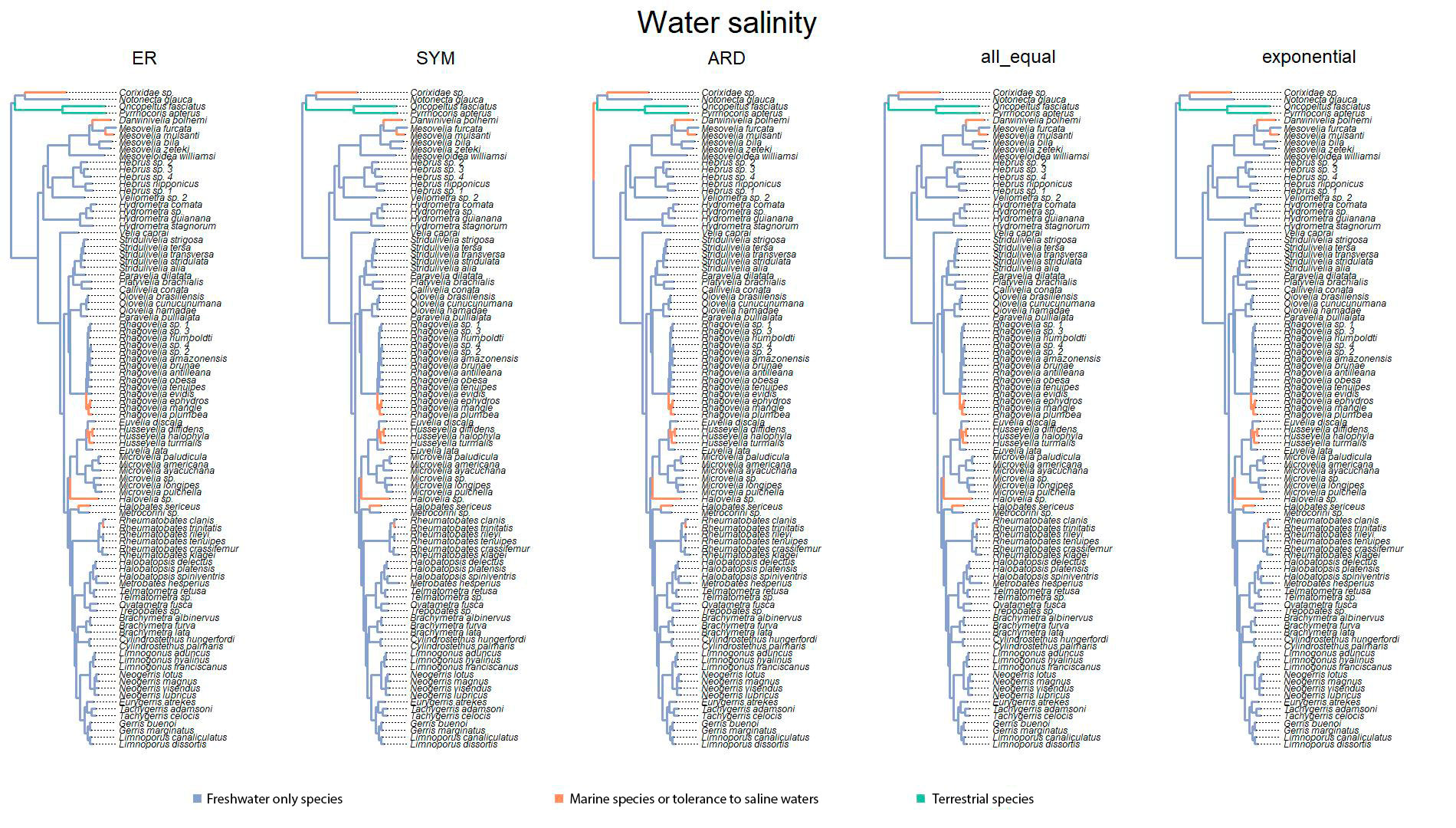

Supplement: msac229_Supplementary_Data [file msac229_supplementary_data.zip › Supplementary_Figure_29.jpg]

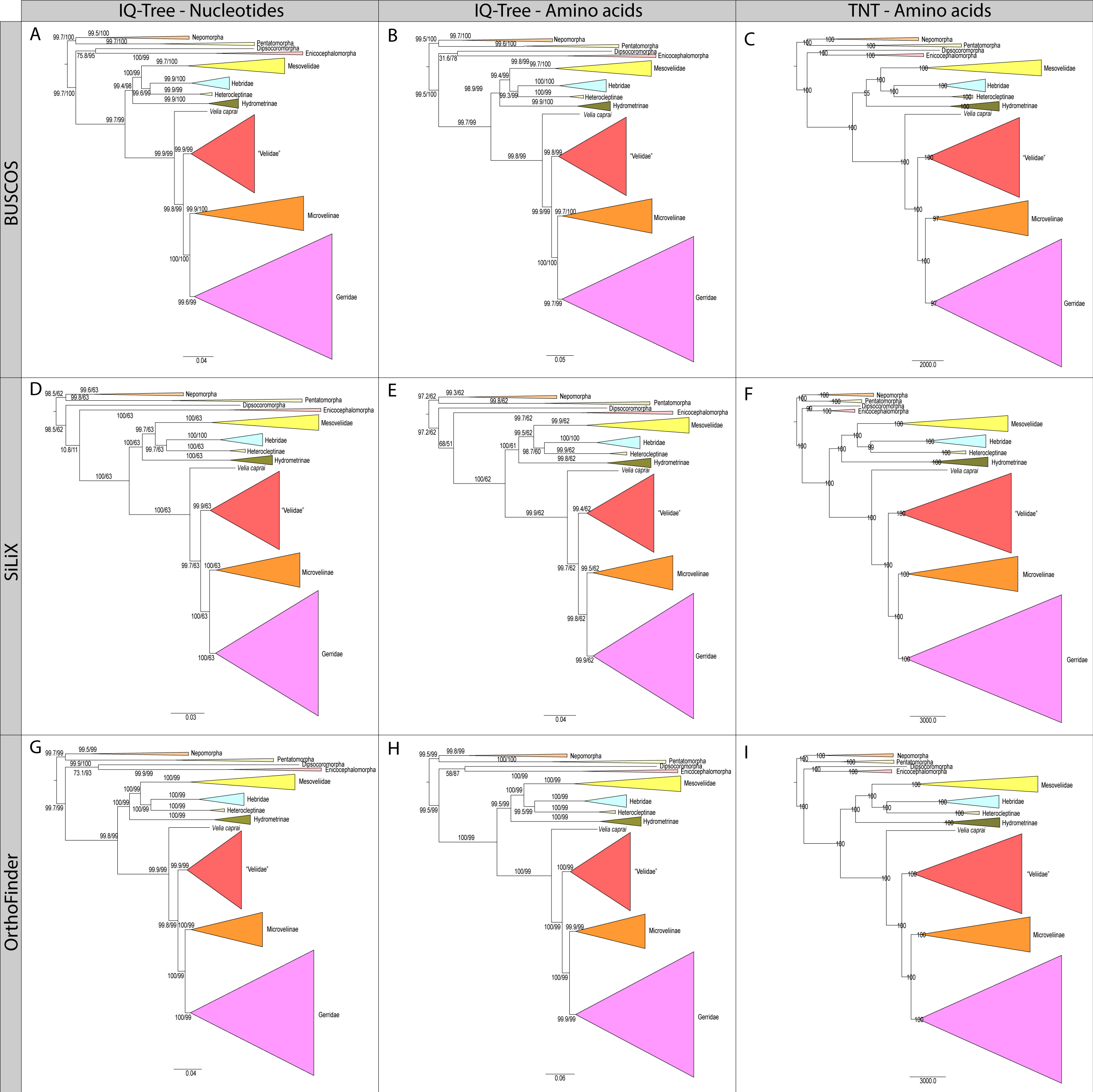

Supplement: msac229_Supplementary_Data [file msac229_supplementary_data.zip › Supplementary_Figure_3.jpg]

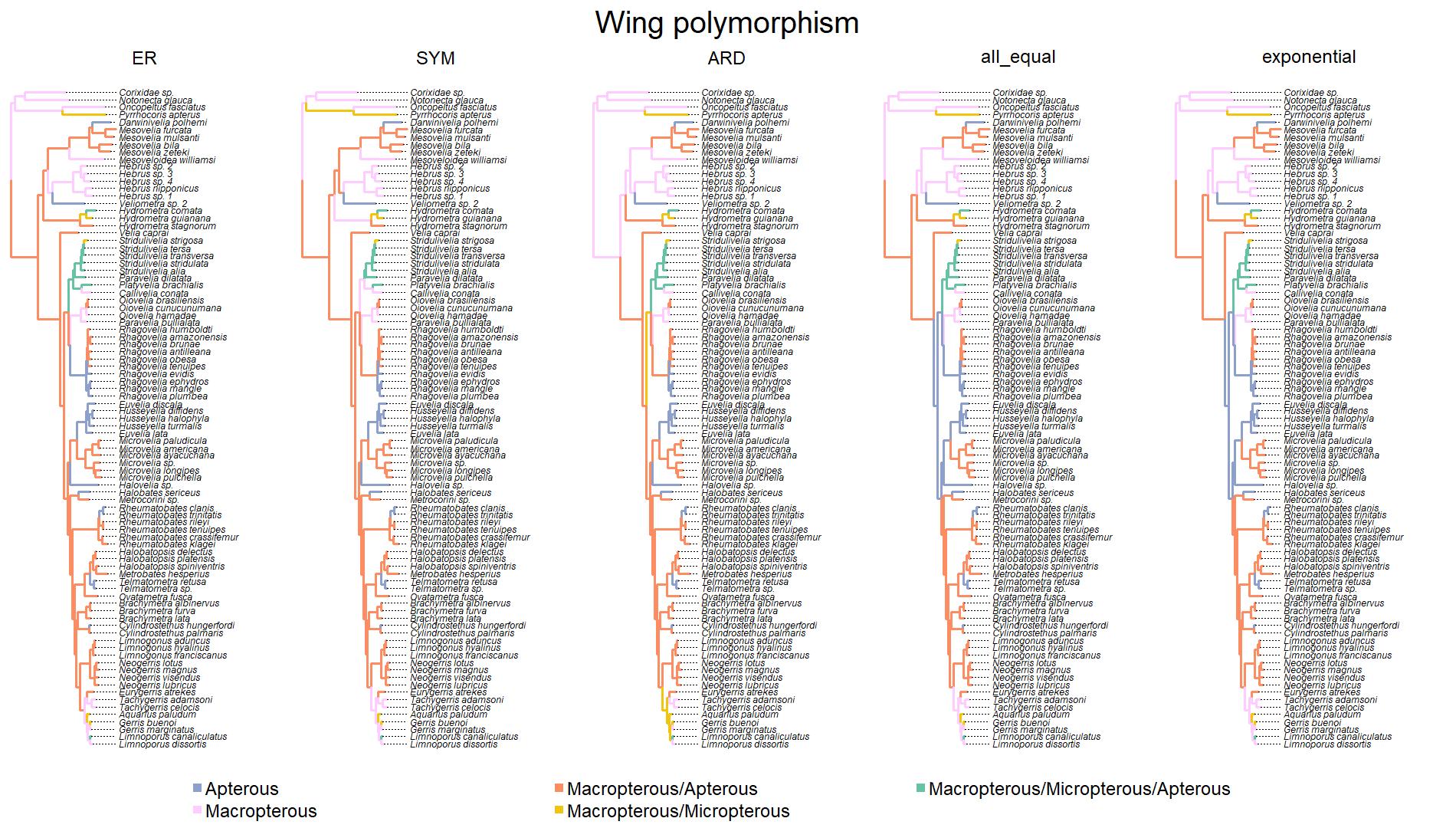

Supplement: msac229_Supplementary_Data [file msac229_supplementary_data.zip › Supplementary_Figure_30.jpg]

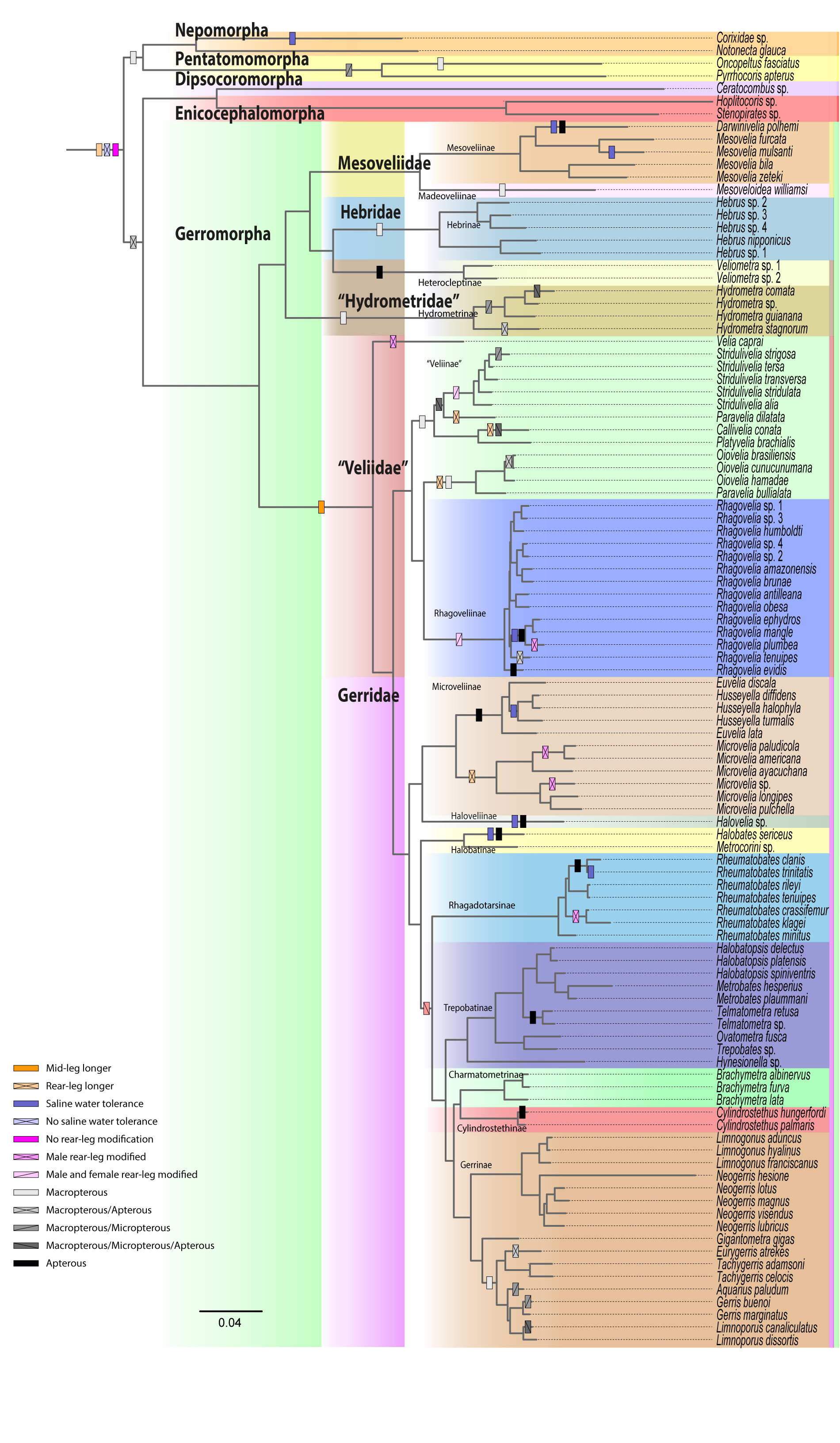

Supplement: msac229_Supplementary_Data [file msac229_supplementary_data.zip › Supplementary_Figure_31.jpg]

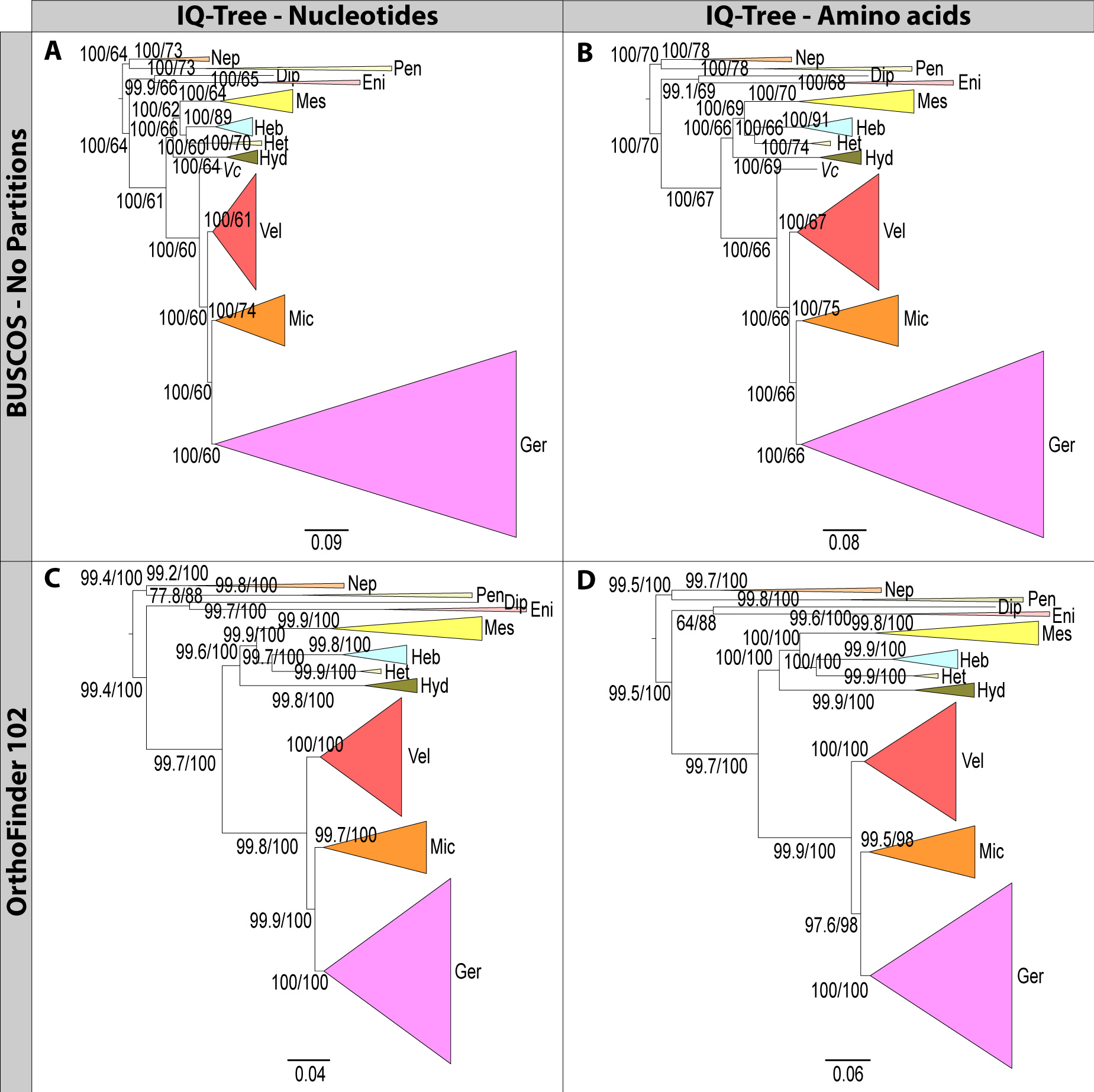

Supplement: msac229_Supplementary_Data [file msac229_supplementary_data.zip › Supplementary_Figure_4.jpg]

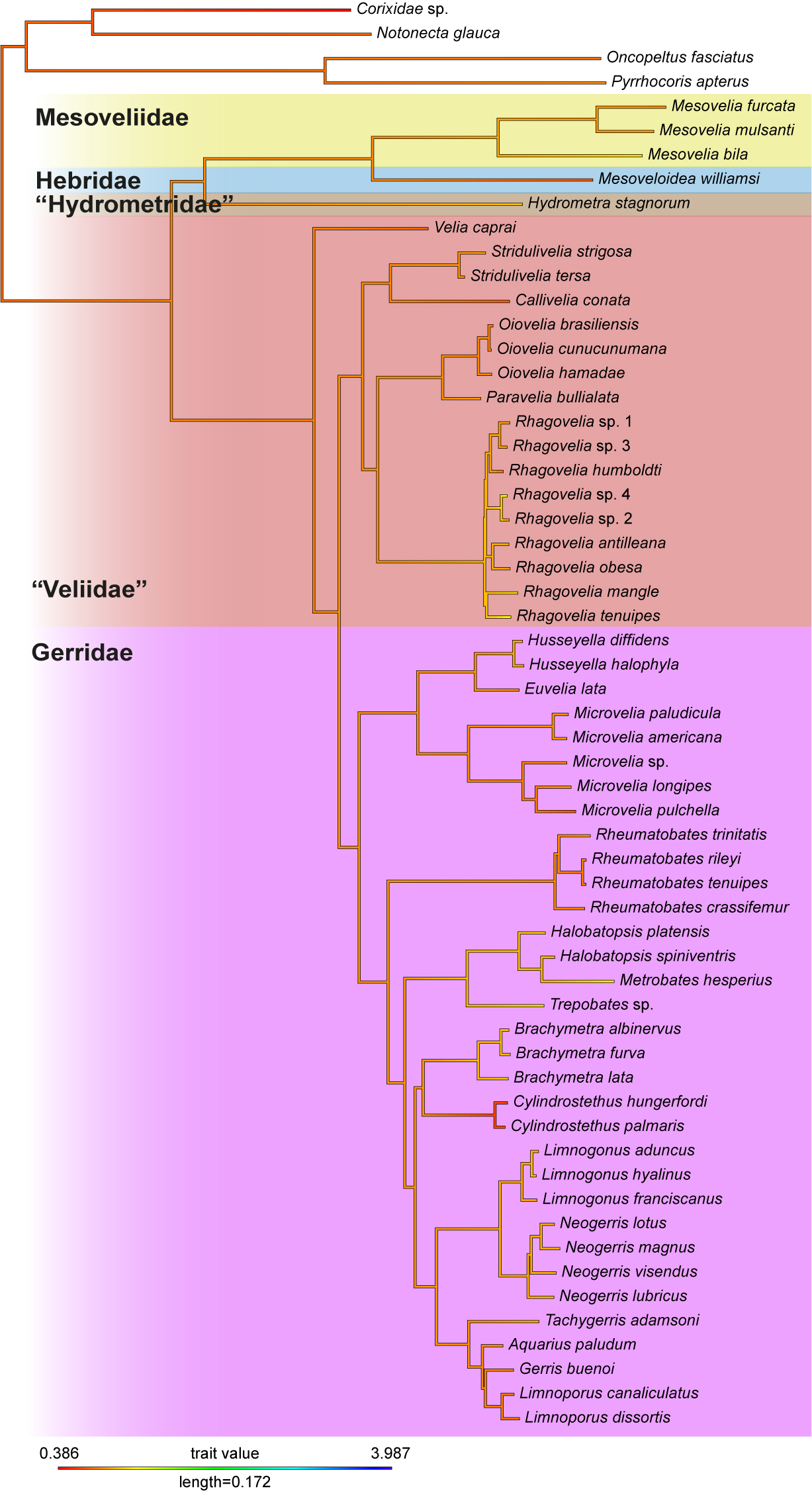

Supplement: msac229_Supplementary_Data [file msac229_supplementary_data.zip › Supplementary_Figure_5.jpg]

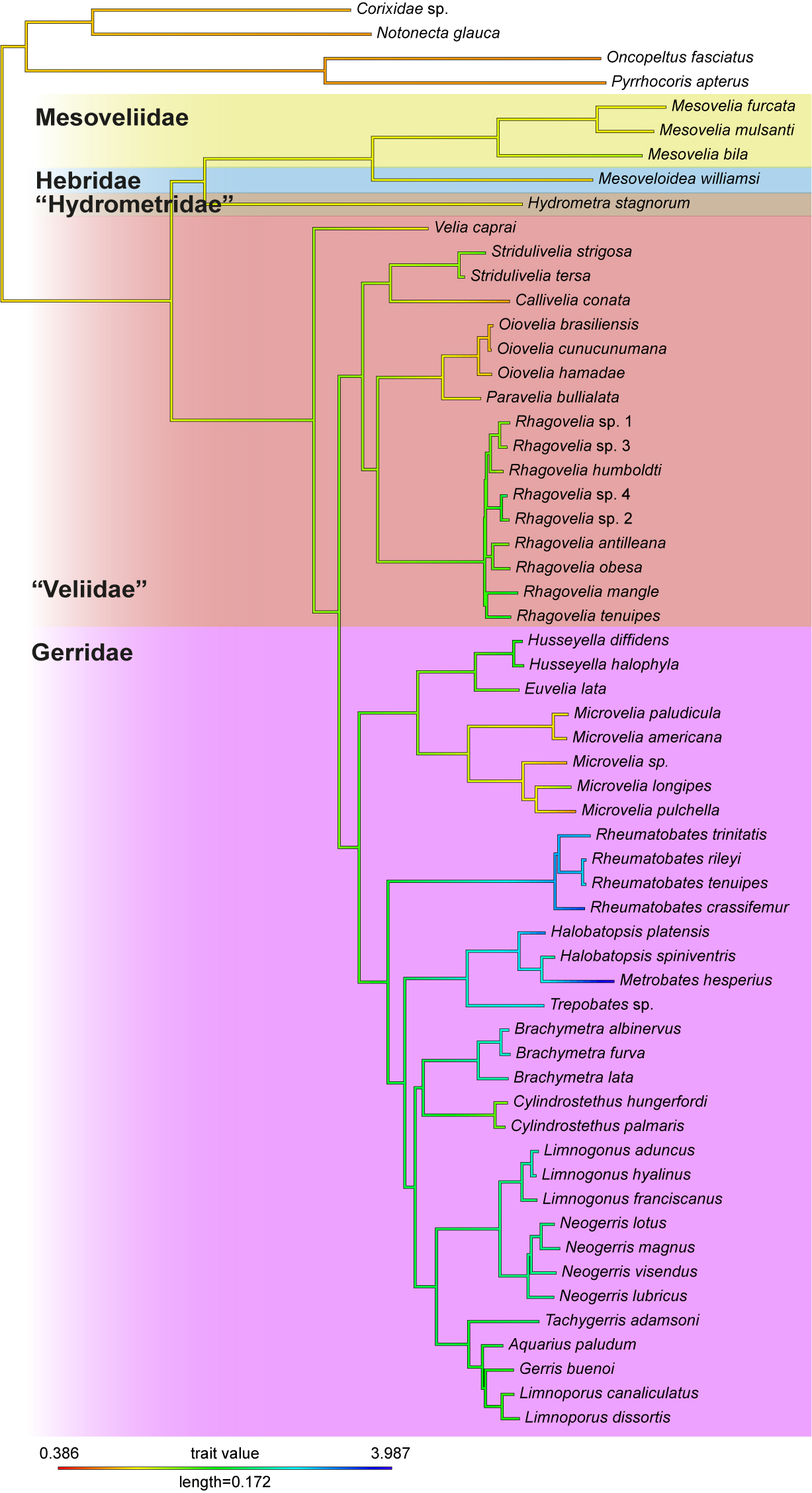

Supplement: msac229_Supplementary_Data [file msac229_supplementary_data.zip › Supplementary_Figure_6.jpg]

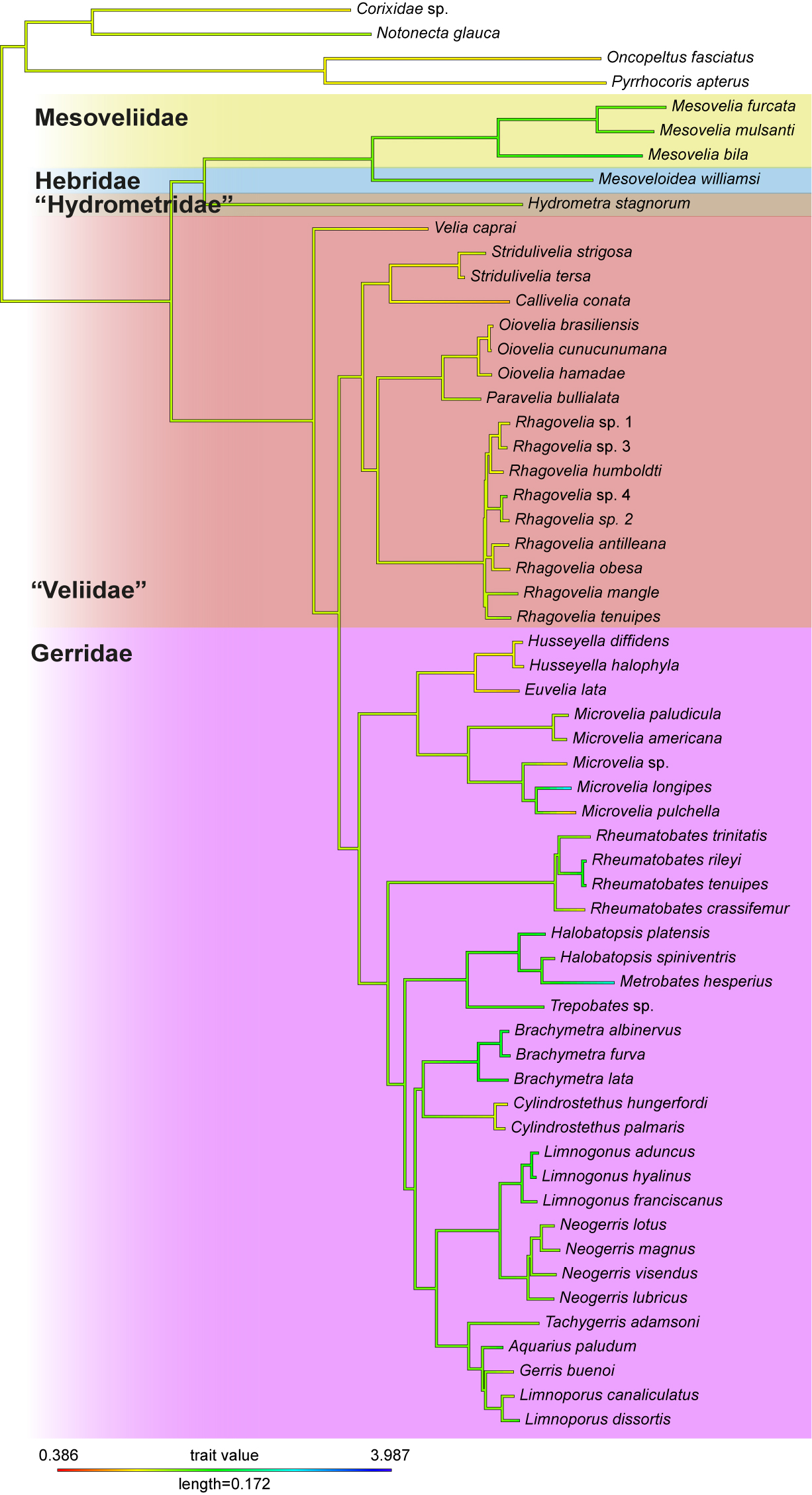

Supplement: msac229_Supplementary_Data [file msac229_supplementary_data.zip › Supplementary_Figure_7.jpg]

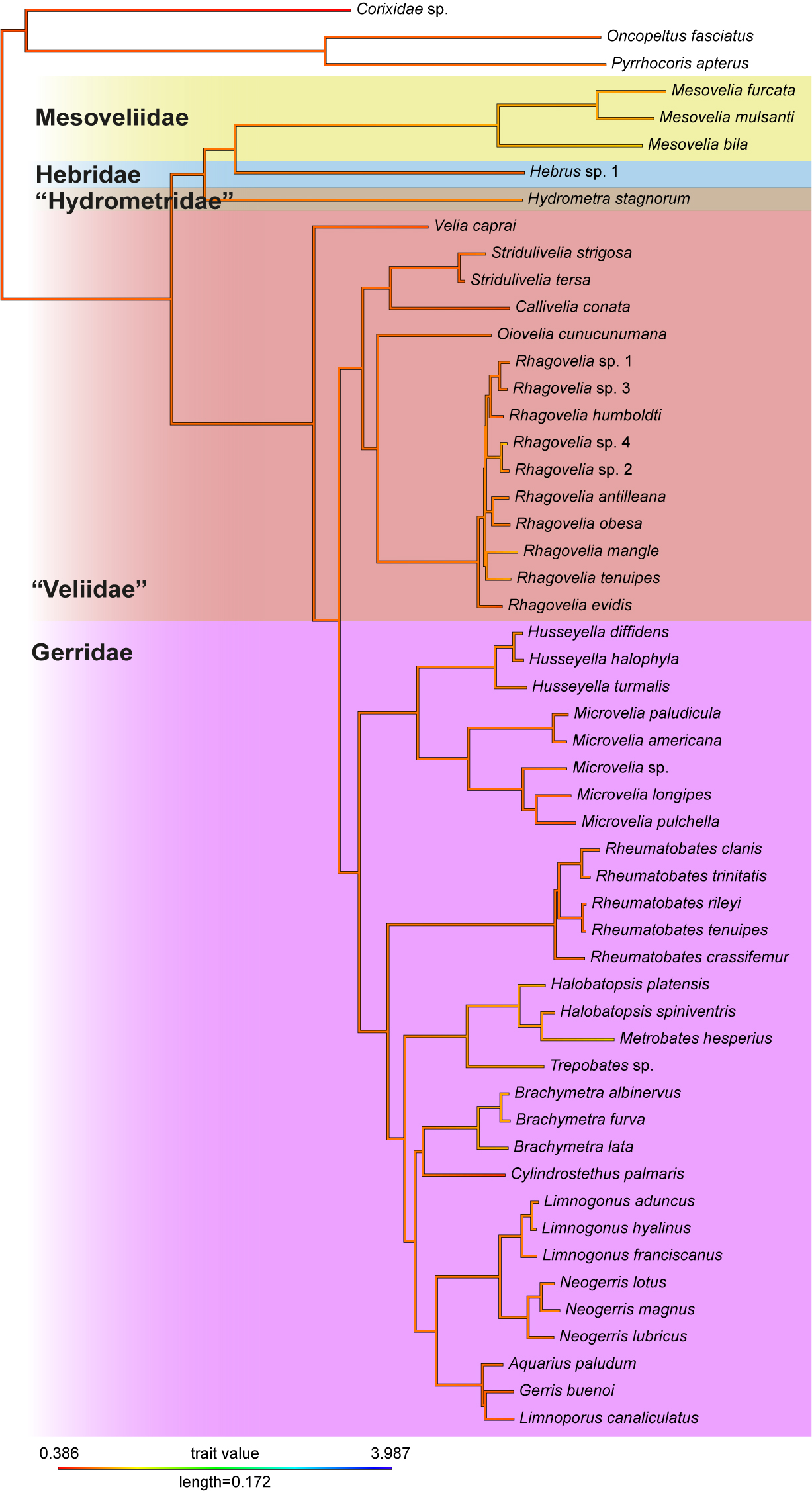

Supplement: msac229_Supplementary_Data [file msac229_supplementary_data.zip › Supplementary_Figure_8.jpg]

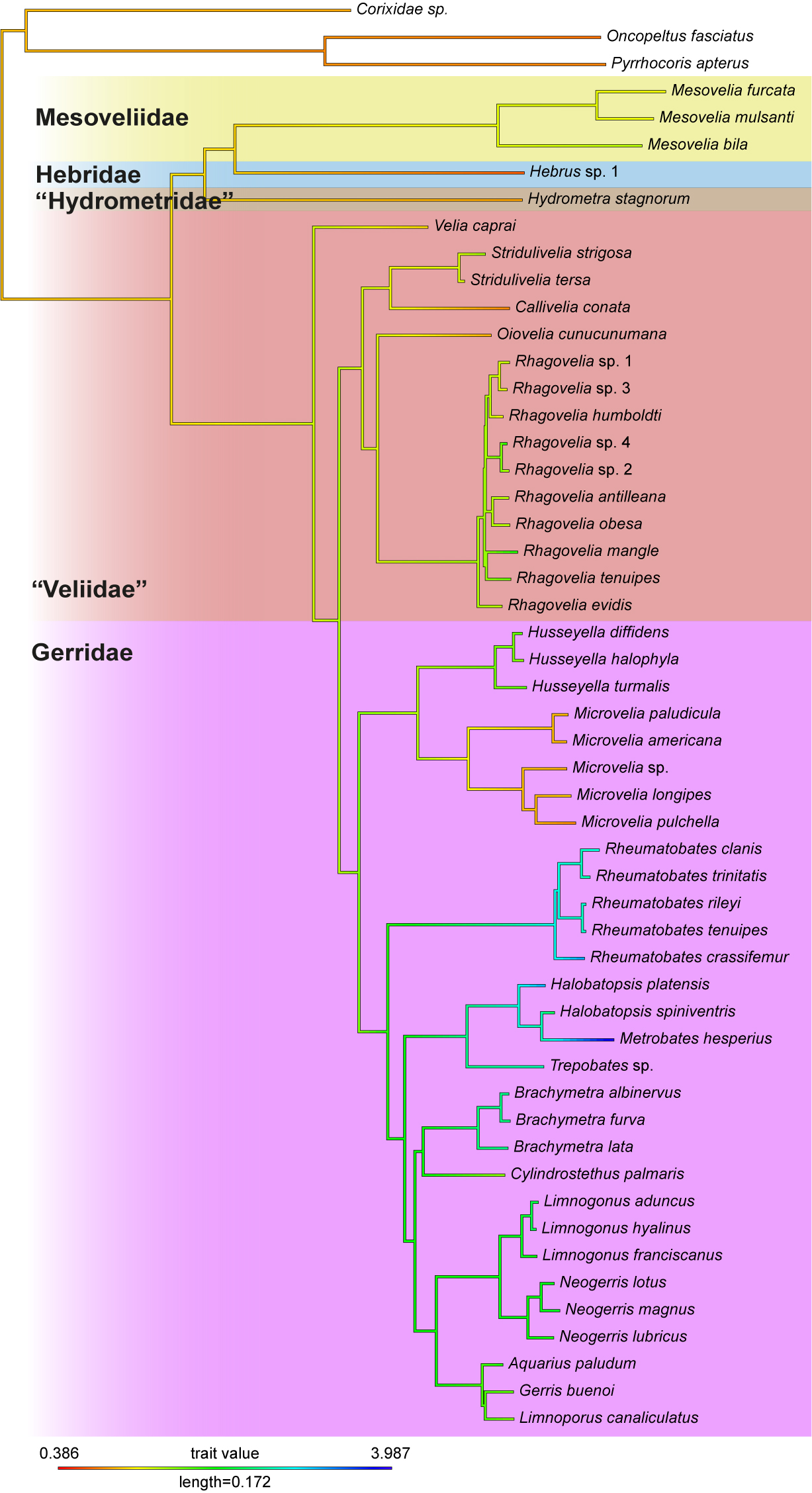

Supplement: msac229_Supplementary_Data [file msac229_supplementary_data.zip › Supplementary_Figure_9.jpg]
